# Deubiquitination of ETV4 by USP7 Promotes NSCLC Tumorigenesis via MAPK7 Activation

**DOI:** 10.1155/humu/9432303

**Published:** 2026-05-06

**Authors:** Xue Meng, Jiaxi Zhang, Ning Zhang, Yuqi Hou, Yimeng Li, Jia Kang, Ruxin Li, Yinghui Shi, Juan Wang, Lixin Cheng, Lingxiao Xing

**Affiliations:** ^1^ Department of Pathology, Hebei Medical University, Shijiazhuang, Hebei, China, hebmu.edu.cn; ^2^ Center of Metabolic Diseases and Cancer Research, Institute of Medical and Health Science, Hebei Medical University, Shijiazhuang, Hebei, China, hebmu.edu.cn; ^3^ Department of Infectious Diseases, Shijiazhuang Fifth Hospital, Shijiazhuang, Hebei, China; ^4^ Department of Geriatrics, Guangdong Provincial Clinical Research Center for Geriatrics, Shenzhen People′s Hospital (The First Affiliated Hospital, Southern University of Science and Technology; The Second Clinical Medical College, Jinan University), Shenzhen, Guangdong, China, jnu.edu.cn; ^5^ Institute of Health Medicine, Southern University of Science and Technology, Shenzhen, Guangdong, China, sustc.edu.cn; ^6^ Department of Pathology, Second Hospital of Hebei Medical University, Shijiazhuang, Hebei, China, hebmu.edu.cn

**Keywords:** deubiquitination, ERK5, ETV4, non–small cell lung cancer, USP7

## Abstract

Transcriptional dysregulation in cancer is accompanied by an anabolic transcriptional response driving proliferation and metabolic adaptation. We previously found that oncogenic ETS variant transcription factor 4 (ETV4) overexpression is associated with DNA replication, glycolytic metabolism, tumor progression, and poor prognosis in non–small cell lung cancer (NSCLC). ETV4 is markedly overexpressed in multiple NSCLC datasets, including TCGA‐LUAD and TCGA‐LUSC. Importantly, ETV4 expression positively correlates with ubiquitin‐specific protease 7 (USP7) and mitogen‐activated protein kinase 7 (MAPK7) levels. While the E3 ligase constitutive photomorphogenesis protein 1 (COP1) is known to regulate ETV4 ubiquitination and degradation, ETV4 deubiquitination remains unclear. Our study reveals that USP7 deubiquitinates ETV4 and protects it from K11‐ and K48‐linked ubiquitination and proteasomal degradation in NSCLC cells. ETV4 transcriptionally controls the expression of the MAPK pathway key gene MAPK7, which encodes extracellular signal‐regulated kinase 5 (ERK5), and participates in the regulation of cell proliferation. Genetic knockdown or pharmacological inhibition of USP7 affects the transcriptional activity of ETV4 on its target gene MAPK7/ERK5. USP7 inhibitor P22077 significantly attenuates ETV4‐MAPK7‐induced cell proliferation in vitro and tumor growth in vivo. Furthermore, elevated ETV4, USP7, and ERK5 protein expressions are associated with poor prognosis of NSCLC patients. These findings identify that USP7 regulates the deubiquitination, stability, and transcriptional activity of ETV4, contributing to the malignant phenotype of ETV4. Inhibition of USP7 might be a promising target in NSCLC with the dysregulation of ETV4 or hyperactivated MAPK signaling.

## 1. Introduction

Gene regulatory programs are major drivers of cellular phenotypes in development and disease and are controlled by sequence‐specific transcription factors (TFs) [1]. The TFs that are deregulated in cancer and have the potential to produce profound changes in gene expression programs fall into three categories: master TFs involved in organizing cell identity (such as OCT4 and TAL1), proliferation control TFs that amplify transcriptional output (such as MYC and TP53), and signaling TFs involved in dynamic changes in the control machinery occurring in response to extracellular signals (such as STAT3 and NOTCH) [2]. These dysregulated transcriptional programs can cause cancer cells to be highly dependent on certain TFs. Improved understanding of dysregulated transcription in cancer cells will be important for the development of effective therapies.

The ETS family of oncogenic TFs is emerging as a crucial mediator of tumorigenesis in solid tumors [3]. We have previously identified that ETS variant transcription factor 4 (ETV4) is the preponderant dysregulated ETS factor associated with glycolytic metabolism, DNA replication, tumor progression, and poor prognosis in non–small cell lung cancer (NSCLC), highlighting the pivotal role of ETV4 in NSCLC development [4–6]. In a stabilized ETV4‐driven murine prostate cancer model, it was demonstrated that high‐dose ETV4 overexpression alone is sufficient to initiate tumorigenesis and functionally synergizes with TP53 loss for tumor progression [7]. In addition, ETV4 is also activated in hepatocellular, gastric, colorectal, and breast cancers [8–11]. A comprehensive bioinformatics evaluation of 28,076 tumor samples spanning 66 distinct types of cancer revealed that ETV4 is one of 568 key cancer driver genes [12]. Furthermore, ETV4 confers resistance to kinase inhibitors (e.g., MAPKi and EGFR‐TKI) and immunotherapies (e.g., lenalidomide and PD‐L1) in multiple cancers [13–16]. Consequently, targeting aberrant ETV4 might represent a promising strategy in cancer therapy. However, TFs have been considered undruggable due to their nuclear localization, structural complexities, and deficiency of prominent binding sites that are often present in kinases or other enzymes [17]. Recent progress in drug discovery and modern chemistry has reignited interest in targeting such proteins directly. It was recently reported that novel ETS factor inhibitors were identified through binding to the conserved ETS DNA‐binding domain (EDBD), which can directly block ETS factor function [18]. However, emerging evidence demonstrated that TFs can exert biological functions independently of their inherent DNA‐binding and transcriptional activation capacities. For example, MYC mutants defective in MAX dimerization or DNA‐binding can still promote cell proliferation (albeit at half the rate that is induced by wild‐type [WT] MYC) [19]. Therefore, an alternative approach to the direct targeting of EDBD is targeting the molecules that are involved in the post‐translational modifications (PTMs), such as ubiquitination, methylation, acetylation, and phosphorylation [20].

Ubiquitination–deubiquitination is the most common PTM, having a key role in regulating protein stability, localization, and activity. Dysregulated ubiquitination of TFs contributes to the hallmark of uncontrolled growth and survival of tumors [21]. For example, tumor suppressor p53 is degraded prematurely due to abnormal ubiquitination, whereas oncogenic TF c‐MYC may gain stability via deubiquitination by preventing its proteasomal degradation, thereby promoting cancer progression [22, 23]. Previous studies have shown that ETV4 protein can be ubiquitinated and degraded by ubiquitin E3 ligase constitutive photomorphogenesis protein 1 (COP1) [24, 25]; however, the deubiquitinating enzymes (DUBs) that regulate ETV4 ubiquitination remain unclear. DUBs reverse ubiquitination, targeting proteins for degradation by proteasomes or lysosomes. DUBs are classified into several families based on their structural and functional similarities, including ubiquitin‐specific proteases (USPs), ubiquitin c‐terminal hydrolases (UCHs), ovarian tumor proteases (OTUs), Machado–Joseph disease protein domain proteases (MJDs), and the JAMM (JAB1/MPN/MOV34) family [26]. Among USPs, USP7 modulates multiple cellular proteins involved in various pathways, including transcription, DNA repair, cell cycle, and epigenetic modification [27–29]. The dysregulation of USP7 in many cancers validates its therapeutic targeting potential. As for NSCLC, USP7 influences cell proliferation, tumor immune evasion, KRAS inhibitor resistance, osimertinib resistance, and cell metabolism [30–34]. However, a comprehensive understanding of the novel substrates by which USP7 regulates NSCLC still needs to be explored.

In this study, we identified that USP7 interacts with ETV4 and maintains ETV4 protein stability via the ubiquitin–proteasome (UPS) pathway in NSCLC cells. ETV4 transcriptionally controls the expression of the MAPK pathway key gene mitogen‐activated protein kinase 7 (MAPK7), which encodes extracellular signal‐regulated kinase 5 (ERK5), and participates in the regulation of cell proliferation of NSCLC cells. Inhibition of USP7 affects the transcriptional activity of ETV4 on its target gene MAPK7/ERK5. USP7 inhibitor P22077 significantly inhibited the proliferation of NSCLC cells and the tumor growth of xenograft tumors induced by ETV4‐MAPK7. Furthermore, elevated ETV4, USP7, and ERK5 protein expressions are associated with poor prognosis of NSCLC patients. Our findings suggest a potential significance of targeting USP7 as a treatment strategy for NSCLC patients exhibiting ETV4 overexpression.

## 2. Materials and Methods

### 2.1. Bioinformatics Analysis

Gene expression data for patients with lung adenocarcinoma (LUAD) and lung squamous cell carcinoma (LUSC) were obtained from the UCSC Xena database [35] (https://xena.ucsc.edu/) and the GEO database. Differential expression analysis was performed using the “limma” R package [36], and genes with adjusted *p* values < 0.01 and |log*F* *C*| > 1 were considered significantly differentially expressed. Functional enrichment analysis of the differentially expressed genes (DEGs) was conducted using the “clusterProfiler” R package to identify significantly enriched biological processes [37]. Potential regulatory TFs for the DEGs were predicted using the KnockTF 2.0 online database [38] (http://www.licpathway.net/KnockTF/index.html). Additionally, interaction data between human deubiquitinases and their potential substrates were downloaded from the UbiBrowser 2.0 database [39] (http://ubibrowser.ncpsb.org.cn) to predict deubiquitinases that may regulate ETV4.

### 2.2. Cell Culture

The human NSCLC cell lines A549, H358, and H1703 were obtained from the China Infrastructure of Cell Line Resources. H1299 was obtained from the Shanghai Institutes for Biological Sciences, Chinese Academy of Sciences. H358T (epithelial–mesenchymal transition‐induced H358 cells with elevated ETV4 expression) [4] and HEK293T were obtained from the laboratory of lipid metabolism of Hebei Medical University. Cells were maintained in RPMI‐1640 or DMEM (10% FBS and 1% penicillin/streptomycin) and incubated at 37°C under 5% CO_2_. USP7 inhibitor P22077 (23704), MG132 (10012628), and cycloheximide (CHX) (14126) were purchased from Cayman Chemical.

### 2.3. siRNA Transfection, Plasmid Construction and Transfection, Generation of Stable Cells, RNA Extraction, cDNA Synthesis, RT‐qPCR, Western Blot Analysis, Chromatin Immunoprecipitation (ChIP) Assay, Luciferase Reporter Assay, and Proximity Ligation Assay (PLA)

Complete methods are available in the Supporting Information section. siRNA sequences and primers used for RT‐qPCR, ChIP‐(q) PCR, and luciferase reporter construction in this study can be found in Supporting Information 4: Table S2.

### 2.4. Label‐Free Quantitative Proteomics Analysis

H1299 cells were lysed, and then the total protein was extracted. Protein complexes were enriched by coimmunoprecipitation (Co‐IP) using anti‐ETV4 antibody (sc‐113, Santa Cruz). LC‐MS/MS and label‐free quantitative proteomics analysis were carried out at Aksomics Technology Co. Ltd. (Shanghai, China). Protein samples were precipitated with TCA, digested with trypsin, and desalted using C18 desalting columns. The obtained peptides were separated using a nano‐UPLC system (EASY‐nLC1200) and analyzed by an online mass spectrometer (Q‐Exactive). Raw LC‐MS/MS data were analyzed with MaxQuant (v1.5.6.0) against the UniProt human database (2016_09) using MS1‐based quantification. The search specified trypsin/P digestion (up to three missed cleavages), carbamidomethylation (C) as a fixed modification, and oxidation (M) and acetylation (N‐term) as variable modifications. The FDR threshold was 0.01 at peptide and protein levels. Quantification was based on unique, unmodified peptides. iBAQ values were also calculated. For the label‐free quantitative proteomics, three independent biological replicates were performed. No imputation was applied; instead, proteins with valid iBAQ values in at least two of three replicates in either group were retained. iBAQ values were log_2_‐transformed prior to fold‐change calculations. Proteins with |log2*F* *C*| > 1 and at least two unique peptides were defined as significantly enriched. Subsequent bioinformatics analyses, including GO functional annotation, KEGG pathway enrichment, and protein–protein interaction network analysis, were performed.

### 2.5. Co‐IP

Following PBS washing, cells were lysed on ice for 30 min in RIPA buffer (3201‐2, BestBio) with protease inhibitors and centrifugation at 16,100 g for 10 min. Ten percent of the supernatant was reserved as input, and the remainder was immunoprecipitated overnight at 4°C using antibodies against ETV4 (sc‐113, Santa Cruz), Flag (14793, CST), HA (sc‐7392, Santa Cruz), USP7 (4833, CST), Ub (1106RM, PTM Bio), or IgG (2729, CST). Protein A/G agarose beads (sc‐2003, Santa Cruz) were added for 2 h. After three washes, bound proteins were eluted in 2× Laemmli buffer, denatured (100°C, 10 min), and separated by SDS‐PAGE.

### 2.6. Cell Proliferation Assays, Clonogenic Assay, EdU Assay for Confocal Microscope Test, and Immunohistochemistry (IHC) Assay

Complete methods are available in the Supporting Information section.

### 2.7. Patients and Clinical Specimens

With approval from Hebei Medical University′s ethics committee, we analyzed formalin‐fixed and paraffin‐embedded (FFPE) sections from 77 lung cancer patients (26 LUSCs and 51 LUADs) diagnosed in the Second or Fourth Hospital of Hebei Medical University between 2017 and 2020. All tissue samples were obtained with informed consent from patients. Patient clinical characteristics are summarized in Supporting Information 3: Table S1. No patients underwent preoperative chemotherapy or radiation therapy. Overall survival was defined as the interval between initial surgery and the death event by 2024.

### 2.8. Animal Studies

All animal procedures were approved by the Experimental Animal Ethics Committee of Hebei Medical University. Five‐week‐old female athymic BALB/c nude mice (Vital River, Beijing, China) were used for animal studies. 5 × 10^6^ A549 NC, A549 sh‐ETV4, or A549 sh‐ETV4+OE ERK5 cells were subcutaneously inoculated into the right flank of each mouse. When tumor volumes reached 100–200 mm^3^, mice received daily intraperitoneal injections of either 20 mg/kg P22077 (23704, Cayman Chemical) or vehicle control (DMSO) for 10 days. Tumor dimensions were measured every 2 days, with volumes calculated using the following formula: tumor volume = 1/2 × (length × width^2^). At termination, mice were humanely euthanized, and tumors were excised, weighed, and processed for histology. Mean tumor weights were statistically compared across experimental groups.

## 3. Statistical Analysis

All quantitative data that follow a normal distribution are presented as mean ± standard deviation (SD). For non‐normally distributed data, including tumor volume, tumor weight, and IHC scores, results are presented as medians with interquartile ranges (IQRs). Statistical analyses were performed using GraphPad Prism 8.3 (GraphPad Software, San Diego, CA) for *t*‐test, one‐way and two‐way analysis of variance (ANOVA), Mann–Whitney *U* test, and Kaplan–Meier analysis. SPSS Statistics Version 26.0 (IBM, Chicago, IL) was used for the *χ* ^2^ test. *p* < 0.05 was considered statistically significant.

## 4. Results

### 4.1. ETV4 Demonstrates a Potential Regulatory Role in NSCLC

**Figure 1:**
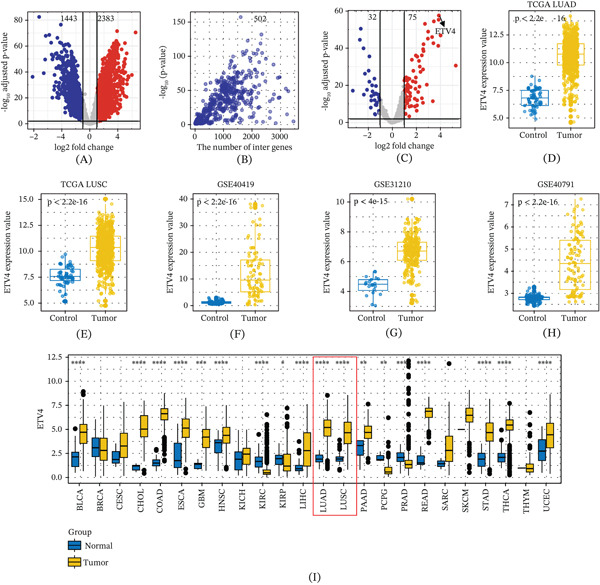
ETV4 is highly expressed in multiple NSCLC datasets. (A) Volcano plot showing differentially expressed genes between LUAD and normal control tissues. Red dots represent upregulated genes, and blue dots represent downregulated genes. (B) Scatter plot illustrating the enrichment of transcription factors for differentially expressed genes. The *x*‐axis represents the number of differentially expressed genes interacting with each transcription factor, and the *y*‐axis represents the enrichment *p* value. (C) Volcano plot displaying differentially expressed transcription factors between LUAD and normal control tissues. (D) Box plot showing the expression levels of ETV4 in LUAD and normal control tissues. (E) Box plot showing the expression levels of ETV4 in LUSC and normal control tissues. (F) Box plot showing the expression levels of ETV4 in the GSE40419 dataset. (G) Box plot showing the expression levels of ETV4 in the GSE31210 dataset. (H) Box plot showing the expression levels of ETV4 in the GSE40791 dataset. (I) Box plot showing the expression levels of ETV4 in the pan‐cancer dataset. ^*^ *p*<0.05, ^**^ *p*<0.01, ^***^ *p*<0.001, and ^****^ *p*<0.0001

Differential expression analysis identified a total of 3826 DEGs in LUAD of the TCGA‐LUAD dataset, including 1443 significantly downregulated genes and 2383 significantly upregulated genes (Figure 1A). Subsequently, TF enrichment analysis of these DEGs was performed using the KnockTF database, revealing 502 potential regulatory TFs (Figure 1B). Among these, 32 TFs were downregulated, and 75 were upregulated (adjusted *p* values < 0.01 and (|log*F* *C*| > log21.2). Notably, ETV4 exhibited the most significant upregulation (Figure 1C). Box plots illustrated the differential expression of ETV4 between normal controls and LUAD samples (Figure 1D). Furthermore, consistent upregulation of ETV4 in lung cancer was observed across additional independent datasets, including TCGA‐LUSC, GSE40419, GSE31210, and GSE40791, highlighting the potential oncogenic importance of ETV4 in lung cancer (Figure 1E–H). Finally, pan‐cancer analysis revealed that ETV4 is upregulated in the majority of cancer types, suggesting that ETV4 may function as a broad‐spectrum oncogene (Figure 1I).

### 4.2. USP7 Interacts With ETV4 and Promotes Its Stability in NSCLC

**Figure 2:**
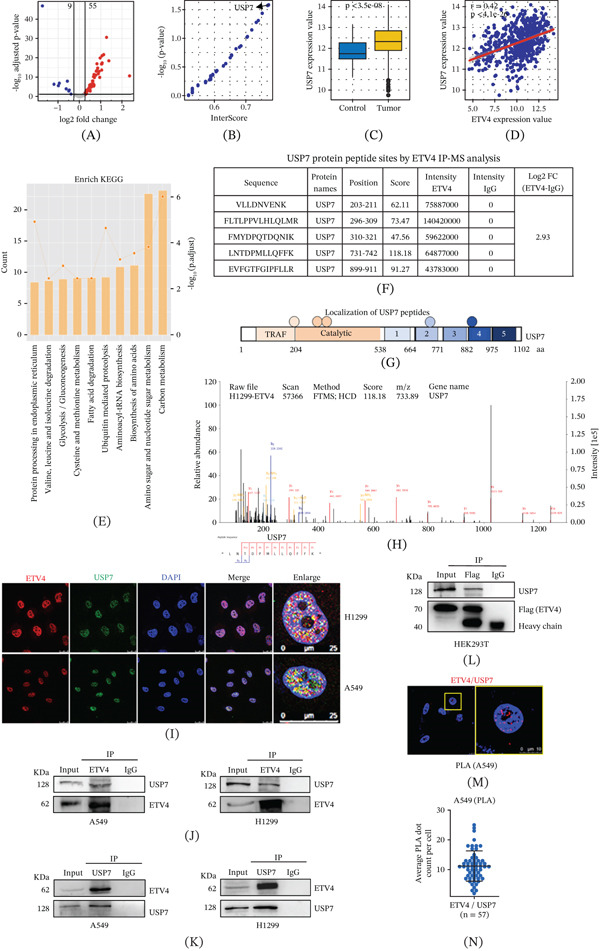
Identification of USP7 as a key deubiquitinase for ETV4. (A) Volcano plot showing differentially expressed deubiquitinases between LUAD and normal control tissues. (B) Scatter plot displaying the confidence scores and *p* values for interactions between each deubiquitinase and ETV4. (C) Box plot showing the expression levels of USP7 in LUAD and normal control tissues. (D) Scatter plot showing the mRNA expression levels of ETV4 and USP7 in individual samples, with the correlation coefficient calculated between ETV4 and USP7 in the TCGA‐LUAD dataset. (E) The Top 10 enriched KEGG pathways of ETV4‐IP proteins in H1299 cells by IP‐proteomics analyses. (F, G) USP7 peptides coimmunoprecipitated with ETV4 in H1299 cells. (H) Representative MS/MS of the USP7 peptides by ETV4‐IP proteomics analyses. (I) Localization of ETV4 and USP7 proteins in A549 and H1299 cells by immunofluorescence staining. (J, K) Reciprocal Co‐IP of endogenous ETV4 and USP7 in A549 and H1299 cells. (L) Co‐IP of exogenous Flag‐ETV4 with USP7 in HEK293T cells using anti‐Flag antibody. (M, N) PLA detection of ETV4‐USP7 interaction (red) in A549 cells. Nuclei were counterstained with DAPI (blue). Scatter plot quantified PLA foci per cell.

Dysregulated ETV4 degradation accelerates lung cancer progression. To identify critical regulators of ETV4 degradation, we investigated the interaction data for human deubiquitinases and their substrates from the UbiBrowser database. This includes 94 deubiquitinases in total, with nine downregulated and 55 upregulated (Figure 2A). Then, we evaluated the interaction scores and *p* values between the ETV4 TF and these differentially expressed deubiquitinases. The results showed that ETV4 had the highest interaction score and the most significant *p* value with USP7 (Figure 2B). Box plots demonstrated the expression differences of USP7 between normal controls and LUAD samples (Figure 2C). Furthermore, correlation analysis revealed a significant positive correlation between ETV4 and USP7 expression (*r* = 0.42), suggesting that USP7 may regulate ETV4 stability through a deubiquitination mechanism (Figure 2D). To validate these findings, IP‐proteomics analysis was performed in H1299 cells using an anti‐ETV4 antibody. There were 751 proteins specifically enriched in ETV4‐IP elutes (FC ≥ 1.5). Top‐enriched KEGG of ETV4‐IP proteins was involved in carbon metabolism, amino sugar and nucleotide sugar metabolism, biosynthesis of amino acids, aminoacyl‐tRNA biosynthesis, ubiquitin‐mediated proteolysis, and fatty acid degradation (Figure 2E). The known ETV4‐interacting proteins, including MCM2‐7, SUPT16H, and ORC1 [6], were also coimmunoprecipitated. Interestingly, the tryptic fragments of USP7 were found to coimmunoprecipitate with ETV4, further suggesting deubiquitinase USP7 as a potential interactor of ETV4. USP7 peptides coimmunoprecipitated with ETV4 were located at the TNF receptor‐associated factor (TRAF), catalytic, and ubiquitin‐like (UBL) domains, respectively (Figure 2F–H). Immunofluorescence experiments indicated colocalization of ETV4 and USP7 in the nucleus of H1299 and A549 cells (Figure 2I), and reciprocal Co‐IP experiments confirmed their interaction (Figure 2J,K). Moreover, IP‐Flag indicated that exogenous ETV4 interacted with USP7 protein in HEK293T cells transfected with Flag‐ETV4 (Figure 2L). PLA further validated the direct interaction, with clear nuclear red foci in A549 cells (Figure 2M,N). These results demonstrate a physical binding between ETV4 and USP7 in NSCLC cells.

### 4.3. USP7 Promotes ETV4 Stabilization via the UPS Pathway

**Figure 3:**
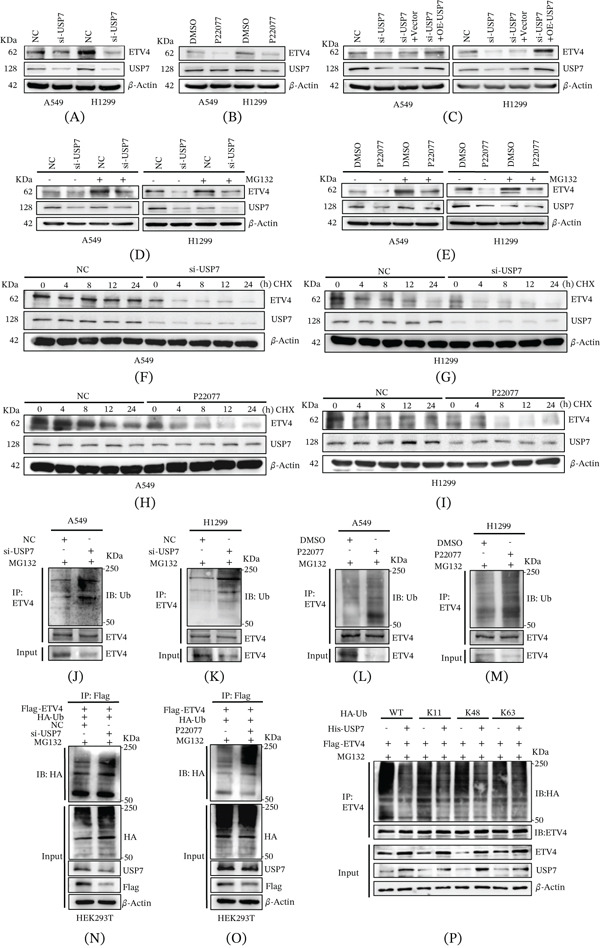
USP7 stabilizes the ETV4 protein by inhibiting the K11‐ and K48‐linked polyubiquitination of ETV4. (A) ETV4 and USP7 protein levels in NSCLC cells transfected with NC or si‐USP7. (B) ETV4 and USP7 protein levels in NSCLC cells treated with 50 *μ*M P22077 for 6 h. (C) Western blot analysis of ETV4 following re‐expression of USP7 in USP7‐knockdown cells. (D, E) ETV4 expression upon USP7 knockdown or inhibition (P22077) in MG132 or solvent control–treated H1299 and A549 cells. All experiments in A–E were repeated three times independently (*n* = 3) using separate batches of cells. The corresponding quantitative analysis is shown in Supporting Information 2: Figure S1A–E. ETV4 stability in cells (F, G) transfected with NC or si‐USP7 or (H, I) treated with solvent control or P22077 (20 *μ*M, 24 h), following treatment with 100 *μ*g/mL CHX for the indicated times. (J–M) Endogenous ETV4 ubiquitination (IP: ETV4, IB: Ub) after USP7 depletion (si‐USP7) or inhibition (P22077, 50 *μ*M, 6 h) in A549 and H1299 cells. (N, O) Exogenous ETV4 ubiquitination (IP: Flag, IB: HA) in HEK293T cells cotransfected with HA‐Ub and Flag‐ETV4, with USP7 knockdown (si‐USP7) or P22077 treatment (50 *μ*M, 6 h). (P) HA‐tagged ubiquitin mutant (WT, K11, K48, and K63) was coexpressed with His‐USP7 and Flag‐ETV4 in HEK293T cells, followed by anti‐HA immunoblot. For all ubiquitination assays presented in J–P, cells were indeed treated with 20 *μ*M MG132 for 6 h before harvest.

To explore whether USP7 influences ETV4 expression, USP7 was genetically depleted using siRNA or pharmacologically inhibited by the irreversible USP7 inhibitor P22077 in A549 and H1299 cells. Western blot results showed that both interventions suppressed ETV4 protein expression (Figure 3A,B), which was restored upon USP7 reintroduction (Figure 3C). To see if ETV4 protein is related to proteasomal degradation, we used the proteasomal inhibitor MG132. The results showed that MG132 treatment elevated ETV4 levels even after ETV4 knockdown (Supporting Information 2: Figure S1F–H) and prevented the reduction caused by USP7 inhibition (Figure 3D,E), suggesting that USP7 buffers proteasome‐dependent ETV4 degradation. Furthermore, upon treatment with protein synthesis inhibitor CHX, both enzymatic activity inhibition and knockdown of USP7 accelerated the degradation of endogenous ETV4 in A549 and H1299 cells (Figure 3F–I and Supporting Information 2: Figure S1F–H). These results indicated that USP7 protects ETV4 stabilization from proteasomal degradation in NSCLC cells.

To further assess the influence of USP7 on ETV4 ubiquitination, USP7 depletion and pharmacological inhibition were performed in A549 and H1299 cells. Both genetic depletion and inhibition of USP7 increased ubiquitination of endogenous ETV4 in A549 and H1299 cells (Figure 3J–M). Similarly, USP7 knockdown or inhibition enhanced ubiquitination of exogenous ETV4 in HEK293T cells cotransfected with HA‐Ub and Flag‐ETV4 (Figure 3N,O), confirming that ETV4 is a bona fide substrate of deubiquitinase USP7. We further explored the ubiquitination linkage pattern of ETV4 using a WT ubiquitin plasmid and three ubiquitin mutants (K11, K48, and K63; all other lysines mutated to arginines). As shown in Figure 3P, USP7 overexpression significantly reduced K11‐ and K48‐linked ubiquitination of ETV4 in HEK293T cells, whereas it had no appreciable effect on the K63‐linked ubiquitination, indicating that K11‐ and K48‐linked ubiquitin chains are involved in ETV4 degradation and are regulated by USP7.

### 4.4. ETV4 Promotes MAPK7 Expression by Binding to the Promoter Region of MAPK7

**Figure 4:**
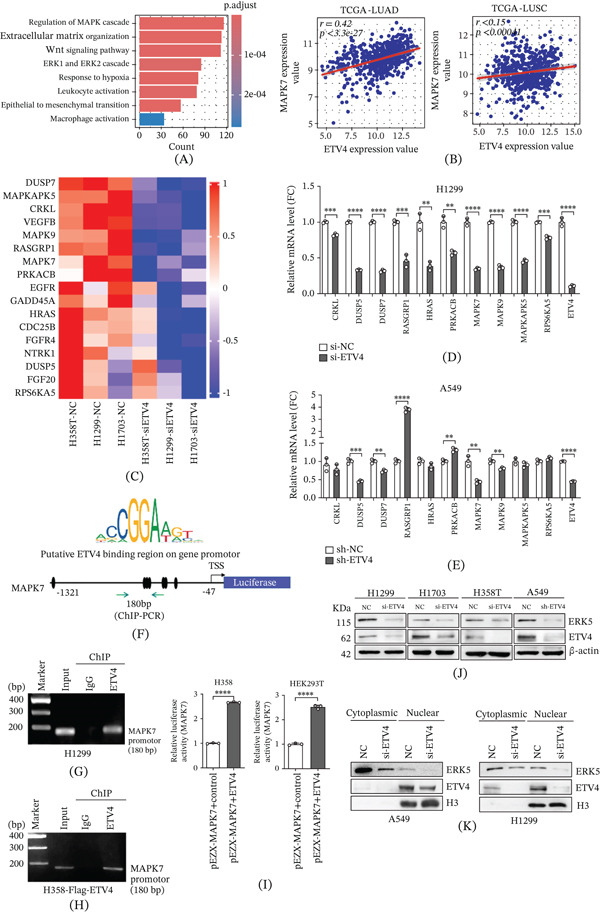
ETV4 regulates MAPK7 expression at the transcriptional level in NSCLC cells. (A) GO enrichment of DEGs in TCGA‐LUAD, highlighting MAPK and Wnt signaling pathway. (B) Positive correlation between ETV4 and MAPK7 expression (*r* = 0.42). (C) Heatmap of downregulated gene signatures (FC > 2, *p* < 0.05) in ETV4‐knockdown NSCLC cells based on human microarray data. (D, E) RT‐qPCR validation of 10 MAPK pathway genes in ETV4‐deficient NSCLC cells. Transcript levels were normalized to ACTB gene expression (mean ± SD, *n* = 3; two‐tailed unpaired *t*‐test).  ^∗∗^ *p* < 0.01,  ^∗∗∗^ *p* < 0.001, and  ^∗∗∗∗^ *p* < 0.0001. (F) Schematic of the putative ETV4 binding region in the MAPK7 promoter, luciferase reporter constructs, and ChIP‐PCR primer locations. (G, H) ChIP‐PCR analysis of ETV4 binding to MAPK7 promoter in endogenous (H1299) and exogenous (Flag‐ETV4 H358) systems. (I) Dual‐luciferase assay of MAPK7 promoter activity in H358 and HEK293T cells cotransfected with reporter and ETV4 or control plasmids (Firefly/Renilla ratio, mean ± SD, *n* = 3; two‐tailed unpaired *t*‐test).  ^∗∗∗∗^ *p* < 0.0001. (J) Western blot analysis of ERK5 (MAPK7) protein expression upon ETV4 knockdown in NSCLC cells. (K) Western blot analysis of ERK5 in subcellular fractions from ETV4‐knockdown H1299 and A549 cells.

To elucidate the molecular mechanism by which ETV4 drives lung cancer progression, we performed functional enrichment analysis on the DEGs previously identified from the TCGA‐LUAD dataset. These DEGs are mainly involved in pathways associated with the activation of the MAPK and Wnt signaling pathways (Figure 4A). Notably, in the TCGA‐LUAD cohort, ETV4 expression exhibited a significant positive correlation with MAPK7 (*r* = 0.42, Figure 4B). Subsequently, we performed microarray analysis on three NSCLC cell lines, H1299, H1703, and H358T, after silencing ETV4. The results showed that 17 genes, including FGF20, FGFR4, EGFR, NTRK1, CRKL, DUSP5, DUSP7, RASGRP1, HRAS, PRKACB, MAPK7, MAPK9, MAPKAPK5, RPS6KA5, CDC25B, GAD45A, and VEGFB, were enriched in the downregulated pathway “MAPK signaling” (Fisher *p* < 0.05, Figure 4C). Among them, we selected 10 genes for further validation. RT‐qPCR results indicated that MAPK7, MAPK9, DUSP5, and DUSP7 were significantly suppressed upon ETV4 knockdown in both transiently (si‐ETV4) and stably (sh‐ETV4) transfected cells (Figure 4D,E and Supporting Information 2: Figure S2A,B). Previous data have reported that ETV4, along with ETV1 and ETV5, is a critical nuclear effector of MAPK signaling [40], and its stability is positively regulated by ERK1/2 activation [41], supporting a potential feedback loop between the ETV4 and MAPK pathway.

Based on the above findings, we focused on MAPK7, as it encodes ERK5, a key molecule in the MAPK pathway. Since ETV4 is a TF, we hypothesize that ETV4 may transcriptionally regulate downstream target genes. To investigate ETV4′s transcriptional regulation of MAPK7, JASPAR was used to predict the ETV4 binding sites in the MAPK7 promoter region (Figure 4F). ChIP assays using both endogenous (anti‐ETV4 in H1299 cells) and exogenous (anti‐Flag in H358 cells) approaches revealed the direct binding of ETV4 to the MAPK7 promoter region (Figure 4G,H). Luciferase reporter assays confirmed the transcriptional activation of the MAPK7 promoter by ETV4 in both H358 and HEK293T cells (Figure 4I). Consistently, ERK5 protein levels decreased upon ETV4 knockdown across multiple NSCLC cell lines (Figure 4J). In addition, to determine whether ETV4 regulates ERK5 expression in a compartment‐specific manner, we performed subcellular fractionation following ETV4 knockdown. Western blot results demonstrated a coordinated decrease of ERK5 levels in both cytoplasmic and nuclear compartments (Figure 4K), suggesting ETV4 modulates total cellular ERK5 abundance rather than its nucleocytoplasmic distribution. These data indicate that ETV4 transcriptionally controls MAPK7 expression in NSCLC cells.

### 4.5. ETV4‐MAPK7 Contributes to Cell Proliferation in NSCLC Cells

To investigate the effects of MAPK7 involved in cell proliferation, we knocked down its expression in H1299, H1703, H358T, and A549 cells using specific siRNA transfection (Supporting Information 2: Figure S3A,B). MAPK7 knockdown reduced cell proliferation (Supporting Information 2: Figure S3C) and colony formation (Supporting Information 2: Figure S3D,E). To determine whether MAPK7 participates in ETV4‐regulated cell proliferation, we established MAPK7‐overexpressing A549‐sh‐ETV4 stable cells (Supporting Information 2: Figure S4A,B). Colony formation assays revealed that MAPK7 overexpression rescued the colony‐forming ability impaired by ETV4 knockdown (Supporting Information 2: Figure S4C,D). And EdU incorporation assays showed that MAPK7 overexpression reversed the proliferation defect caused by ETV4 knockdown, as evidenced by restored EdU incorporation (Supporting Information 2: Figure S4E,F). The above results demonstrated that MAPK7 is a critical downstream effector of ETV4 in sustaining NSCLC proliferation.

### 4.6. USP7 Modulates ETV4‐Dependent Transcriptional Regulation of MAPK7

**Figure 5:**
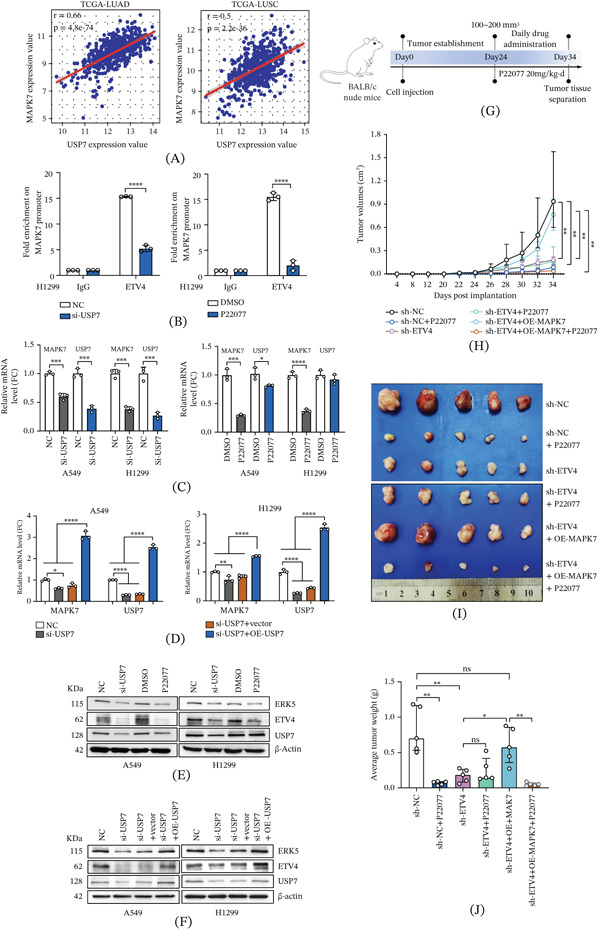
USP7 modulates ETV4‐dependent transcriptional regulation of MAPK7. (A) Significant positive correlation between USP7 and MAPK7 expression in the TCGA‐LUAD and TCGA‐LUSC datasets. (B) ChIP‐qPCR assessment of ETV4 enrichment at the MAPK7 promoter region under conditions of USP7 depletion or inhibition in H1299 cells (mean ± SD, *n* = 3; two‐tailed unpaired *t*‐test).  ^∗∗∗∗^ *p* < 0.0001. (C) RT‐qPCR analysis of MAPK7 and USP7 expression in NSCLC cells after USP7 knockdown (si‐USP7) or pharmacological inhibition (P22077, 50 *μ*M, 6 h) (mean ± SD, *n* = 3; two‐tailed unpaired *t*‐test).  ^∗^ *p* < 0.05,  ^∗∗∗^ *p* < 0.001, and  ^∗∗∗∗^ *p* < 0.0001. (D) RT‐qPCR analysis of MAPK7 and USP7 transcript levels under USP7 knockdown and rescue conditions, with normalization to ACTB expression (mean ± SD; *n* = 3; one‐way ANOVA followed by Tukey′s multiple comparisons test).  ^∗^ *p* < 0.05,  ^∗∗^ *p* < 0.01, and  ^∗∗∗∗^ *p* < 0.0001. (E) Western blot analysis of ETV4 and ERK5 protein expression following genetic or pharmacological USP7 suppression in multiple NSCLC cell lines. (F) Western blot detection of ETV4 and ERK5 protein levels upon USP7 reconstitution in USP7‐deficient cells. (G) Schematic diagram of the animal studies. (H) Tumor growth curves of A549‐derived xenografts following P22077 administration (20 mg/kg/day, intraperitoneal injection, 10 days; data are presented as median (IQR), *n* = 5 per group. Each comparison between the two groups at endpoint was performed using the Mann–Whitney *U* test.  ^∗∗^ *p* < 0.01. (I) Representative tumors images excised at endpoint (Day 34). (J) Tumor weights at sacrifice are shown as median (IQR), *n* = 5. Each comparison was conducted using the Mann–Whitney *U* test. ns, not significant;  ^∗^ *p* < 0.05 and  ^∗∗^ *p* < 0.01.

In the TCGA‐LUAD and LUSC datasets, we found a significant correlation between USP7 and MAPK7 expression (Figure 5A). ETV4 protein expression changed correspondingly with USP7 knockdown or overexpression. Critically, ChIP‐qPCR assays indicated that knockdown or pharmacological inhibition of USP7 significantly reduced ETV4 binding at the MAPK7 promoter (Figure 5B). To determine whether USP7 affects ETV4‐mediated transcriptional activation of MAPK7, we first detected the effects of USP7 inhibition on MAPK7 expression. qRT‐PCR analysis indicated that USP7 knockdown or inhibition (Figure 5C and Supporting Information 2: Figure S5A) significantly reduced MAPK7 mRNA levels in A549, H1299, H1703, and H358T cells. These inhibitory effects were reversed by USP7 re‐expression (Figure 5D and Supporting Information 2: Figure S5B). Consistently, Western blot analysis revealed that USP7 knockdown or inhibition reduced ERK5 protein levels, which were also restored upon USP7 reconstitution (Figure 5E,F and Supporting Information 2: Figure S5C,D). These results demonstrated that USP7‐mediated stabilization of ETV4 is essential for its transcriptional regulation of MAPK7.

To investigate the effects of USP7 on NSCLC cell proliferation, MTT assays were conducted in H1299, H1703, H358T, and A549 cells following P22077 treatment. As shown in Supporting Information 2: Figure S5E–H, treatment with P22077 significantly suppressed the cell proliferation of NSCLC cells. Notably, P22077 inhibited cell proliferation in sh‐NC and sh‐ETV4+OE‐MAPK7 cells, but not in sh‐ETV4 cells (Supporting Information 2: Figure S5I). In vivo, xenograft models from these cell lines were established, and each model was then divided into vehicle control (DMSO) and P22077‐treated groups (Figure 5G). As shown in Figure 5H–J, ETV4 knockdown markedly reduced the tumor growth rate, size, and weight compared to sh‐NC cells (sh‐ETV4 vs. sh‐NC, *p* < 0.01), whereas overexpression of MAPK7 reversed the inhibitory effects of ETV4 deletion (sh‐ETV4+OE‐MAPK7 vs. sh‐ETV4, *p* < 0.05), indicating the role of ETV4‐MAPK7 on tumor growth. Compared to the vehicle control group, P22077 treatment significantly inhibited the tumor growth of sh‐NC and sh‐ETV4+OE‐MAPK7 groups, whereas it did not show the synergistic effects on tumor growth in the sh‐ETV4 group. These findings indicate that USP7 inhibition attenuates cell proliferation and tumor growth by targeting the ETV4‐MAPK7 axis in an ETV4‐dependent manner.

### 4.7. Elevated ETV4, USP7, and ERK5 Protein Expressions Are Associated With Poor Prognosis of NSCLCs

**Figure 6:**
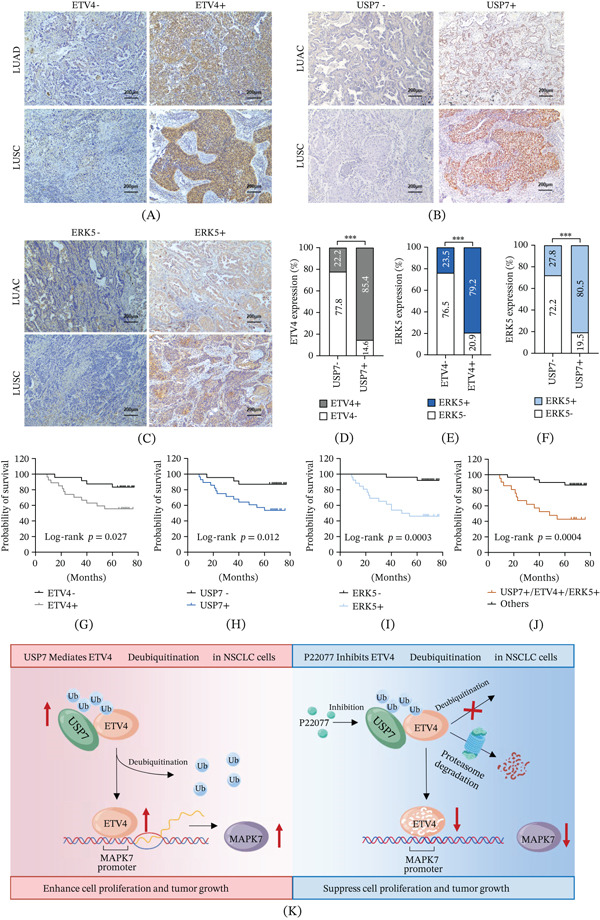
Elevated ETV4, USP7, and ERK5 protein expressions are associated with poor prognosis of NSCLCs. (A–C) Representative IHC images of ETV4, USP7, and ERK5 in LUAD and LUSC tissues (scale bar, 200 *μ*m). (D–F) Correlation analysis of USP7 with ETV4, USP7 with ERK5, and ETV4 with ERK5 expression from 77 patients based on *χ* ^2^ test.  ^∗∗∗^ *p* < 0.001. (G–J) Kaplan–Meier survival analysis of 51 LUAD patients based on the expression level of ETV4, USP7, or ERK5. Log‐rank (Mantel–Cox) test was used to calculate *p* values. (K) USP7 stabilizes ETV4 to drive ETV4‐MAPK7‐induced cell proliferation and tumor growth in NSCLC.

To explore the clinical relevance of ETV4, USP7, and ERK5 in NSCLCs, we performed IHC on FFPE sections from 77 patients. Positive nuclear ETV4 staining was detected in 43 of 77 (55.84%) tumor specimens and significantly correlated with larger tumor size, lymph node metastasis, distant metastasis, and advanced TNM stage (Figure 6A; Supporting Information 3: Table S1, all *p* < 0.01). Nuclear staining of USP7 was positive in 53.25% (41 of 77) cases and associated with increased tumor size and clinical stage (Figure 6B; Supporting Information 3: Table S1, all *p* < 0.05). Cytoplasmic ERK5 expression was observed in 54.55% (42 of 77) of tumor tissues and correlated with tumor size, metastasis, and clinical stage (Figure 6C; Supporting Information 3: Table S1, all *p* < 0.05). As indicated in Figure 6D–F, strong correlations were observed between USP7 and ETV4 expression (*r* = 0.634, *p* < 0.001), USP7 and ERK5 expression (*r* = 0.554, *p* < 0.001), and ETV4 and ERK5 expression (*r* = 0.514, *p* < 0.001). Besides, the Kaplan–Meier survival analysis demonstrated that high expression of ETV4 (log‐rank *p* = 0.027), USP7 (log‐rank *p* = 0.012), ERK5 (log‐rank *p* < 0.001), or ETV4+/USP7+/ERK5+ coexpression (log‐rank *p* < 0.001) was significantly associated with poorer overall survival (Figure 6G–J). Thus, elevated ETV4, USP7, and ERK5 expression could be valuable biomarkers for predicting aggressive disease progression and unfavorable clinical outcomes in NSCLCs.

## 5. Discussion

Pan‐cancer analysis has demonstrated that ETV4 dysregulation occurs across multiple malignancies, and its overexpression is related to tumor progression, poor prognosis, immune subtypes, and patient drug sensitivity, suggesting that ETV4 may serve as a critical therapeutic target or predictive biomarker for multiple cancers [42, 43]. In this study, we identified that the deubiquitinase USP7 interacts with ETV4 and protects it from UPS‐mediated degradation in NSCLC (Figure 6K). USP7 inhibition disrupted ETV4‐mediated transcriptional activation of its target gene, MAPK7, and suppressed ETV4‐MAPK7‐induced cell proliferation in vitro and tumor growth in vivo, indicating that USP7 targeting might be a promising strategy for ETV4‐overexpressing lung cancer.

TFs, such as c‐Jun, ETV4, and ETV5, are often regulated via UPS degradation, which is a critical mechanism enabling rapid transcriptional changes during cellular stress [44]. The E3 ubiquitin ligase COP1 acts as a tumor suppressor by targeting oncogenic substrates, including JUN, p53, MTA1, and PEA3 family TFs (ETV1/4/5), for degradation via the UPS system [24, 45–49]. Post‐translational regulation of ETS by COP1 is also critical for lung branching morphogenesis [25], pancreatic *β*‐cell insulin secretion [50], and mouse brain development [51]. However, the specific deubiquitinase (DUB) counteracting ETV4 ubiquitination remained unknown. Our study reveals USP7 as the first DUB that binds to, deubiquitinates, and stabilizes ETV4. USP7 is a well‐characterized regulator of tumorigenesis and cancer progression, exerting its oncogenic activity primarily by stabilizing key tumor‐promoting substrates through deubiquitination, including KRAS [32], MYC [52], *β*‐catenin [53], and ETS2 [54]. Here, our IP‐proteomics analyses, Co‐IP, and PLA results demonstrated the physical interaction between USP7 and ETV4. Furthermore, pharmacological inhibition or genetic ablation of USP7 led to a marked reduction in ETV4 protein levels, while USP7 reintroduction restored ETV4 expression, validating a specific USP7‐ETV4 regulatory axis. COP1 is established as the E3 ubiquitin ligase for ETV4, and USP7 has also been shown to stabilize ETV4, so the two proteins appear to exert opposing effects on ETV4 stability. However, it remains unknown whether USP7 and COP1 are functionally associated or whether they merely act as independent regulators of ETV4. This leads to an intriguing hypothesis: whether, under specific spatiotemporal conditions, the two proteins can be recruited into the same protein complex, thereby regulating the dynamic balance of ETV4. This is also a question we plan to explore in future work.

Through deubiquitinase activity, USP7 modulates the stability, function, and subcellular localization of the target proteins. Distinct ubiquitination patterns lead to different outcomes. The K48‐linked polyubiquitin chains represent the canonical signal for proteasomal degradation [55]. K63‐linkage facilitates the autophagic degradation of protein substrates or modulates nondegradative processes such as cellular signaling, intracellular trafficking, DNA damage response, and other contexts [56]. K11‐linkage, an atypical type of ubiquitination, also generally functions as a primary proteasomal degron [56]. It has been reported that the hydrolyzing activity of USP7 on the ubiquitin chain of K6, K11, K48, and K63 is significantly better than that of K27 and K29 [57]. In our study, we found that USP7 cleaves K11‐ and K48‐linked polyubiquitin chains from ETV4 but exhibits no activity toward K63‐linked chains. This mechanism aligns with previous reports that USP7 binds to Raf‐1 and decreases its K11‐ and K48‐linked polyubiquitination, regulating the ERK1/2 signaling pathway in LUAD [58]. USP7 binds to LRRK2 and protects it from UPS degradation by deubiquitinating K48‐linked polyubiquitin chains in acute myeloid leukemia [59]. A recent study also indicated that USP7 is responsible for removing the K48‐linked polyubiquitination signal from the transcription factor EB (TFEB), thereby preventing its proteasomal degradation, and provides the foundation for therapeutic targeting of the USP7‐TFEB axis in certain cancers characterized by TFEB dysregulation [60]. The preference of USP7 for specific ubiquitin chains may depend on both its structural features and the substrate′s ubiquitination pattern. Structurally, USP7 contains a TRAF‐like domain (aa 62–205), a catalytic domain (aa 208–560), and a UBL region (aa 562–1083) [61]. The TRAF and UBL domains mediate substrate recognition and binding, while the catalytic domain′s hydrolysis activity toward distinct ubiquitin chains is influenced by UBL‐ or substrate‐induced conformational changes. Which domain of USP7 interacts with ETV4 is still under investigation in our laboratory. Regarding the ubiquitination pattern, K11‐linked chains are often found in a branched chain with K48, which enhances protein degradation by the proteasome [56]. However, whether ETV4 carries K11/K48 branched chains, or whether K63‐linked chains exist at other sites that are sterically inaccessible to USP7, remains unknown. High‐resolution structural information on the ubiquitin linkage types on ETV4 is still lacking and warrants further study. Collectively, we indicated that USP7 removes the K11‐ and K48‐linked polyubiquitin of ETV4 to protect it from proteasomal degradation, unveiling a possible mechanism by which USP7 governs ETV4 stability in NSCLC.

The MAPK cascade signaling transduces extracellular stimuli into diverse intracellular responses, including transcriptional reprogramming. One of the best characterized modes is the MAPK ERK1/2 signaling/PEA3‐ETS protein stability axis, which dynamically couples ERK activation to transcriptional output through COP1‐mediated ubiquitination and degradation of ETS proteins [37, 62], positioning ETV4 as a critical effector downstream of MAPK signaling. More interestingly, our microarray analyses identified a common set of genes enriched in “MAPK signaling” in NSCLC, including the key negative‐feedback modulators of the RAS/MAPK pathway, DUSP5 and DUSP7, implying a potential role of ETV4 in maintaining MAPK homeostasis. Furthermore, we found that MAPK7/ERK5 is transcriptionally regulated by ETV4, acting as a critical downstream effector of ETV4 in sustaining NSCLC proliferation. ERK5, which possesses a unique C‐terminal extension distinct from other MAPK family members [63], represents a newly discovered MAPK signaling pathway that mediates resistance to BRAF, MEK1/2, and ERK1/2 inhibitors through activation of the MEK5/ERK5 cascade [64]. It has been reported that MAPK7/ERK5 expression or its kinase activity is critical for tumorigenesis. For example, MAPK7 silencing reduced the invasion and metastasis ability of breast cancer in vitro and in vivo [65]. In lung cancer, elevated MAPK7 mRNA and ERK5 protein levels correlate with tumor progression [66], and MEK5/ERK5 co‐overexpression predicts poor survival in LUAD. CRISPR/Cas9‐mediated ERK5 ablation not only attenuates tumor growth but also enhances its sensitivity to conventional therapies [67]. Collectively, ETV4 might be a regulatory node that couples ERK1/2 and ERK5 MAPK signaling, although the precise feedback mechanisms remain to be further elucidated.

Accumulating evidence demonstrated that dysregulated expression and activation of USP7 are closely associated with tumor progression and poor prognosis across multiple cancer types, including NSCLC [31, 68]. Preclinical studies have demonstrated that USP7 inhibitors P22077 and P5091 exhibit antiproliferative effects in NSCLC models and enhance sensitivity to paclitaxel, a PARP inhibitor, and PRMT1‐based therapy [12, 69–71]. Our research demonstrates that P22077 reduces the stability of the ETV4 protein by inhibiting the DUB activity of USP7, thereby lowering the level of ETV4 protein. As a TF, ETV4 directly regulates the transcriptional expression of MAPK7. Therefore, treatment with P22077 leads to a decrease in ETV4 levels, which in turn suppresses the expression of MAPK7 and ultimately inhibits the proliferation of tumor cells. In the sh‐ETV4 group, since ETV4 has been stably knocked down, the key effector molecule of P22077 (i.e., ETV4 protein) is largely absent, resulting in a relatively weak inhibitory effect of P22077 on the proliferation of sh‐ETV4 cells, with almost no impact on tumor growth. It is expected that in the sh‐ETV4+OE‐MAPK7 group, where ETV4 is knocked down and MAPK7 is exogenously overexpressed, due to the low expression level of ETV4, P22077 should also be unable to effectively inhibit tumor growth. However, our experimental results show that in sh‐ETV4+OE‐MAPK7 cells, overexpression of MAPK7 leads to a significant recovery of ETV4 expression levels (Supporting Information 2: Figure S4B). This phenomenon indicates that MAPK7 may positively regulate the expression of ETV4 through some feedback mechanism, thereby restoring the sensitivity of sh‐ETV4+OE‐MAPK7 cells to P22077. Therefore, P22077 treatment effectively inhibited the tumorigenic ability of sh‐ETV4+OE‐MAPK7 cells, even resulting in a smaller tumor volume than the sh‐ETV4+P22077 group. These findings suggest that overexpression of MAPK7 not only may directly promote proliferation but also enhance its tumor‐promoting effect by upregulating ETV4 expression, further highlighting the key regulatory role of the ETV4‐MAPK7 axis in NSCLC. Interestingly, we found that MAPK7 overexpression restored ETV4 protein levels in A549 sh‐ETV4 cells without increasing its mRNA (Supporting Information 2: Figure S4A), indicating a post‐translational regulation. It is well established that activated ERK1/2 signaling stabilizes ETV4 by anchoring COP1 to the nuclear membrane. We speculate that ERK5 (encoded by MAPK7), which shares structural similarity with ERK1/2, may stabilize ETV4 via a similar mechanism, creating a positive feedback loop. Admittedly, our data have not validated whether ERK5 kinase activity is involved, because ERK5 protein levels do not equate to pathway activation. Nevertheless, overexpression alone may confer basal activity and produce partial effects. Such results support the potential therapeutic value of USP7 inhibitors in NSCLC with ETV4 overexpression. USP7 inhibitor‐mediated ETV4 reduction decreases MAPK7 expression, which in turn further lowers ETV4. This amplified inhibitory effect may allow USP7 inhibitors to achieve effective ETV4 suppression at lower doses, thereby widening the therapeutic window. Conversely, if tumors harbor MAPK7 overexpression or ERK5 activation, the positive feedback could rapidly restore ETV4 levels and drive resistance, thereby requiring a higher USP7 inhibitor dosage or ERK5‐targeted combination therapy to recover the window. USP7, ETV4, and ERK5 expression may serve as potential biomarkers to predict the poor prognosis of NSCLCs.

In conclusion, we discovered that USP7 is a previously unrecognized deubiquitinase that interacts with ETV4 and catalyzes the removal of polyubiquitin chains to sustain the oncogene fixation in NSCLC. P22077 potently suppressed tumor growth induced by ETV4‐MAPK7, and this inhibitory effect was dependent on substrate availability. Therefore, USP7 may be a novel therapeutic target for NSCLC characterized by ETV4 dysregulation or hyperactivated MAPK signaling.

**Nomenclature**
CHX: cycloheximide
COP1: constitutive photomorphogenesis protein 1
DUB: deubiquitinating enzyme
ETV4: ETS variant transcription factor 4
ERK5: extracellular signal‐regulated kinase 5
LUAD: lung adenocarcinoma
LUSC: lung squamous cell carcinoma
MAPK7: mitogen‐activated protein kinase 7
NSCLC: non–small cell lung cancer
PLA: proximity ligation assay
PTMs: post‐translational modifications
TF: transcription factor
UPS: ubiquitin–proteasome
USP7: ubiquitin‐specific protease 7

## Funding

This study was funded by the Natural Science Foundation of Hebei Province (10.13039/501100003787) (H2023206434).

## Ethics Statement

The animal studies were approved by the Experimental Animal Ethics Committee, Hebei Medical University (IACUC‐Hebmu‐2023017). All patients involved in this study provided informed consent.

## Conflicts of Interest

The authors declare no conflicts of interest.

## Supporting Information

Additional supporting information can be found online in the Supporting Information section. The supporting information includes a detailed experimental protocol.

## Supporting information


**Supporting Information 1** Supporting materials and methods.

**Supporting Information 2** Additional results (Figures S1–S5).

**Supporting Information 3** Table S1 showing relationships between ETV4, USP7, and ERK5 expression and clinicopathologic parameters.

**Supporting Information 4** Table S2 listing siRNA and primer sequences.

## Data Availability

All data are included in the article or in the Supporting Information section.

## References

[1] How P. M. , Eukaryotic Transcriptional Activators Work, Nature. (1988) 335, no. 6192, 683–689, 10.1038/335683a0, 2-s2.0-0023683667. 3050531

[2] Bradner J. E. , Hnisz D. , and Young R. A. , Transcriptional Addiction in Cancer, Cell. (2017) 168, no. 4, 629–643, 10.1016/j.cell.2016.12.013, 2-s2.0-85012194101, 28187285. 28187285 PMC5308559

[3] Sizemore G. M. , Pitarresi J. R. , Balakrishnan S. , and Ostrowski M. C. , The ETS Family of Oncogenic Transcription Factors in Solid Tumours, Nature Reviews Cancer. (2017) 17, no. 6, 337–351, 10.1038/nrc.2017.20, 2-s2.0-85019263601. 28450705

[4] Wang Y. , Ding X. , Liu B. , Li M. , Chang Y. , Shen H. , Xie S. M. , Xing L. , and Li Y. , ETV4 Overexpression Promotes Progression of Non–Small Cell Lung Cancer by Upregulating PXN and MMP1 Transcriptionally, Molecular Carcinogenesis. (2020) 59, no. 1, 73–86, 10.1002/mc.23130, 31670855. 31670855

[5] Liu B. , Zhang J. , Meng X. , Xie S. M. , Liu F. , Chen H. , Yao D. , Li M. , Guo M. , Shen H. , Zhang X. , and Xing L. , HDAC6-G3BP2 Promotes Lysosomal-TSC2 and Suppresses mTORC1 Under ETV4 Targeting-Induced Low-Lactate Stress in Non-Small Cell Lung Cancer, Oncogene. (2023) 42, no. 15, 1181–1195, 10.1038/s41388-023-02641-6, 36823378. 36823378

[6] Zhang J. , Wang Y. , Cao S. , Xie S. M. , Liu B. , Li Y. , Hou Y. , Meng X. , Ruan M. , Bu D. , Kang J. , Li R. , Lou L. , Wang J. , and Xing L. , Topoisomerase I Inhibition in ETV4-Overexpressed Non-Small Cell Lung Cancer Promotes Replication and Transcription Mediated R-Loop Accumulation and DNA Damage, Advanced Science. (2025) 12, no. 35, 10.1002/advs.202409307, 40548893. PMC12462987 40548893

[7] Li D. , Zhan Y. , Wang N. , Tang F. , Lee C. J. , Bayshtok G. , Moore A. R. , Wong E. W. P. , Pachai M. R. , Xie Y. , Sher J. , Zhao J. L. , Khudoynazarova M. , Gopalan A. , Chan J. , Khurana E. , Shepherd P. , Navone N. M. , Chi P. , and Chen Y. , ETV4 Mediates Dosage-Dependent Prostate Tumor Initiation and Cooperates With p53 Loss to Generate Prostate cancer, Advances. (2023) 9, no. 14, 10.1126/sciadv.adc9446, 37018402. PMC10075989 37018402

[8] Xie M. , Lin Z. , Ji X. , Luo X. , Zhang Z. , Sun M. , Chen X. , Zhang B. , Liang H. , Liu D. , Feng Y. , Wang Y. , Li Y. , Liu B. , Huang W. , and Xia L. , FGF19/FGFR4-Mediated Elevation of ETV4 Facilitates Hepatocellular Carcinoma Metastasis by Upregulating PD-L1 and CCL2, Journal of Hepatology. (2023) 79, no. 1, 109–125, 10.1016/j.jhep.2023.02.036, 36907560. 36907560

[9] Keld R. , Guo B. , Downey P. , Cummins R. , Gulmann C. , Ang Y. S. , and Sharrocks A. D. , PEA3/ETV4-Related Transcription Factors Coupled With Active ERK Signalling Are Associated With Poor Prognosis in Gastric Adenocarcinoma, British Journal of Cancer. (2011) 105, no. 1, 124–130, 10.1038/bjc.2011.187, 2-s2.0-79959742995, 21673681. 21673681 PMC3137405

[10] Zhang X. , He Y. , Shen J. , Zhou B. , Qin H. , Zhang S. , and Huang Z. , Study on the Mechanism of FOXA2 Activation on Glutathione Metabolic Reprogramming Mediated by ETV4 Transcription to Facilitate Colorectal Cancer Malignant Progression, Biochemical Genetics. (2025) 63, no. 5, 4179–4199, 10.1007/s10528-024-10918-y, 39316306. 39316306

[11] Zhu T. , Zheng J. , Zhuo W. , Pan P. , Li M. , Zhang W. , Zhou H. , Gao Y. , Li X. , and Liu Z. , ETV4 Promotes Breast Cancer Cell Stemness by Activating Glycolysis and CXCR4-Mediated Sonic Hedgehog signaling, Discovery. (2021) 7, no. 1, 10.1038/s41420-021-00508-x, 34052833. PMC8164634 34052833

[12] Martínez-Jiménez F. , Muiños F. , Sentís I. , Deu-Pons J. , Reyes-Salazar I. , Arnedo-Pac C. , Mularoni L. , Pich O. , Bonet J. , Kranas H. , Gonzalez-Perez A. , and Lopez-Bigas N. , A Compendium of Mutational Cancer Driver Genes, Nature Reviews Cancer. (2020) 20, no. 10, 555–572, 10.1038/s41568-020-0290-x, 32778778. 32778778

[13] Wang B. , Krall E. B. , Aguirre A. J. , Kim M. , Widlund H. R. , Doshi M. B. , Sicinska E. , Sulahian R. , Goodale A. , Cowley G. S. , Piccioni F. , Doench J. G. , Root D. E. , and Hahn W. C. , ATXN1L, CIC, and ETS Transcription Factors Modulate Sensitivity to MAPK Pathway Inhibition, Cell Reports. (2017) 18, no. 6, 1543–1557, 10.1016/j.celrep.2017.01.031, 2-s2.0-85011933931, 28178529. 28178529 PMC5313047

[14] Liao S. , Davoli T. , Leng Y. , Li M. Z. , Xu Q. , and Elledge S. J. , A Genetic Interaction Analysis Identifies Cancer Drivers That Modify EGFR Dependency, Genes and Development. (2017) 31, no. 2, 184–196, 10.1101/gad.291948.116, 2-s2.0-85014075511, 28167502. 28167502 PMC5322732

[15] Neri P. , Barwick B. G. , Jung D. , Patton J. C. , Maity R. , Tagoug I. , Stein C. K. , Tilmont R. , Leblay N. , Ahn S. , Lee H. , Welsh S. J. , Riggs D. L. , Stong N. , Flynt E. , Thakurta A. , Keats J. J. , Lonial S. , Bergsagel P. L. , Boise L. H. , and Bahlis N. J. , ETV4-Dependent Transcriptional Plasticity Maintains MYC Expression and Results in IMiD Resistance in Multiple Myeloma, Blood Cancer Discovery. (2024) 5, no. 1, 56–73, 10.1158/2643-3230.bcd-23-0061, 37934799. 37934799 PMC10772538

[16] Ma P. , Jin X. , Fan Z. , Wang Z. , Yue S. , Wu C. , Chen S. , Wu Y. , Chen M. , Gu D. , Zhang S. , Mao R. , and Fan Y. , Super-Enhancer Receives Signals From the Extracellular Matrix to Induce PD-L1-Mediated Immune Evasion via Integrin/BRAF/TAK1/ERK/ETV4 Signaling, Cancer Biology and Medicine. (2021) 19, no. 5, 669–684, 10.20892/j.issn.2095-3941.2021.0137, 34623791. 34623791 PMC9196059

[17] Xie X. , Yu T. , Li X. , Zhang N. , Foster L. J. , Peng C. , Huang W. , and He G. , Recent Advances in Targeting the “Undruggable” Proteins: From Drug Discovery to Clinical trials, Therapy. (2023) 8, no. 1, 10.1038/s41392-023-01589-z, 37669923. PMC10480221 37669923

[18] Abdalla S. , Forghany Z. , Ma J. , Hollander J. G. , Nachane R. , Szuhai K. , Hogendoorn P. C. W. , ten Dijke P. , Shah D. , and Baker D. A. , Identification of Novel Small Molecule Inhibitors of ETS Transcription Factors, FEBS Letters. (2025) 599, no. 12, 1733–1748, 10.1002/1873-3468.70040, 40214124. 40214124 PMC12183613

[19] Cowling V. H. and Cole M. D. , The Myc Transactivation Domain Promotes Global Phosphorylation of the RNA Polymerase II Carboxy-Terminal Domain Independently of Direct DNA Binding, Molecular and Cellular Biology. (2007) 27, no. 6, 2059–2073, 10.1128/mcb.01828-06, 2-s2.0-33947285072, 17242204. 17242204 PMC1820498

[20] Qian M. , Yan F. , Yuan T. , Yang B. , He Q. , and Zhu H. , Targeting Post-Translational Modification of Transcription Factors as Cancer Therapy, Drug Discovery Today. (2020) 25, no. 8, 1502–1512, 10.1016/j.drudis.2020.06.005. 32540433

[21] Kim D. , Nam H. J. , and Baek S. H. , Ubiquitination of Transcription Factors in Cancer: Unveiling Therapeutic Potential, Molecular Oncology. (2025) 19, no. 8, 2174–2195, 10.1002/1878-0261.70033. 40227962 PMC12330937

[22] Zhao K. , Yang Y. , Zhang G. , Wang C. , Wang D. , Wu M. , and Mei Y. , Regulation of the Mdm2–p53 Pathway by the Ubiquitin E3 ligaseMARCH7, EMBO Reports. (2018) 19, no. 2, 305–319, 10.15252/embr.201744465, 2-s2.0-85039798047, 29295817. 29295817 PMC5797962

[23] Shin H. , Hwang S. , Jeong J. H. , Shin S. C. , Oh Y. , Kim J. , Hwang I. , Kim E. E. K. , Choo H. , and Song E. J. , Targeting USP47 Enhances the Efficacy of KRAS Inhibitor in KRASG12C Mutated Non-Small Cell Lung Cancer by Controlling Deubiquitination of c-Myc, Pharmacological Research. (2025) 215, 107722, 10.1016/j.phrs.2025.107722, 40180254. 40180254

[24] Vitari A. C. , Leong K. G. , Newton K. , Yee C. , O’Rourke K. , Liu J. , Phu L. , Vij R. , Ferrando R. , Couto S. S. , Mohan S. , Pandita A. , Hongo J. A. , Arnott D. , Wertz I. E. , Gao W. Q. , French D. M. , and Dixit V. M. , COP1 Is a Tumour Suppressor That Causes Degradation of ETS Transcription Factors, Nature. (2011) 474, no. 7351, 403–406, 10.1038/nature10005, 2-s2.0-79959192993, 21572435. 21572435

[25] Zhang Y. , Yokoyama S. , Herriges J. C. , Zhang Z. , Young R. E. , Verheyden J. M. , and Sun X. , E3 Ubiquitin Ligase RFWD2 Controls Lung Branching Through Protein-Level Regulation of ETV Transcription Factors, Proceedings of the National Academy of Sciences. (2016) 113, no. 27, 7557–7562, 10.1073/pnas.1603310113, 2-s2.0-84977279455, 27335464. PMC4941481 27335464

[26] Guan X. , Wang Y. , Yu W. , Wei Y. , Lu Y. , Dai E. , Dong X. , Zhao B. , Hu C. , Yuan L. , Luan X. , Miao K. , Chen B. , Cheng X. D. , Zhang W. , and Qin J. J. , Blocking Ubiquitin-Specific Protease 7 Induces Ferroptosis in Gastric Cancer via Targeting Stearoyl-CoA Desaturase, Science. (2024) 11, no. 18, 10.1002/advs.202307899, 38460164. PMC11095140 38460164

[27] Pozhidaeva A. and Bezsonova I. , USP7: Structure, Substrate Specificity, and Inhibition, DNA Repair. (2019) 76, 30–39, 10.1016/j.dnarep.2019.02.005, 2-s2.0-85061858373, 30807924. 30807924 PMC6481172

[28] Nininahazwe L. , Liu B. , He C. , Zhang H. , and Chen Z. S. , The Emerging Nature of Ubiquitin-Specific Protease 7 (USP7): A New Target in Cancer Therapy, Drug Discovery Today. (2021) 26, no. 2, 490–502, 10.1016/j.drudis.2020.10.028. 33157193

[29] Zhou J. , Wang J. , Chen C. , Yuan H. , Wen X. , and Sun H. , USP7: Target Validation and Drug Discovery for Cancer Therapy, Medicinal Chemistry. (2018) 14, no. 1, 10.2174/1573406413666171020115539, 2-s2.0-85041671850. 29065837

[30] Li J. , Han Y. , Zhang H. , Qian Z. , Jia W. , Gao Y. , Zheng H. , and Li B. , The m6A Demethylase FTO Promotes the Growth of Lung Cancer Cells by Regulating the m6A Level of USP7 mRNA, Biochemical and Biophysical Research Communications.(2019) 512, no. 3, 479–485, 10.1016/j.bbrc.2019.03.093, 2-s2.0-85064478146, 30905413. 30905413

[31] Yu Z. Z. , Liu Y. Y. , Zhu W. , Xiao D. , Huang W. , Lu S. S. , Yi H. , Zeng T. , Feng X. P. , Yuan L. , Qiu J. Y. , Wu D. , Wen Q. , Zhou J. H. , Zhuang W. , and Xiao Z. Q. , ANXA1-Derived Peptide for Targeting PD-L1 Degradation Inhibits Tumor Immune Evasion in Multiple Cancers, Journal for Immuno Therapy of Cancer. (2023) 11, no. 3, e006345, 10.1136/jitc-2022-006345, 37001908. PMC10069584 37001908

[32] Huang B. , Cao D. , Yuan X. , Xiong Y. , Chen B. , Wang Y. , Niu X. , Tian R. , and Huang H. , USP7 Deubiquitinates KRAS and Promotes Non-Small Cell Lung Cancer, Cell Reports. (2024) 43, no. 11, 114917, 10.1016/j.celrep.2024.114917, 39499616. 39499616

[33] Meng Y. , Lin W. , Wang N. , Wei X. , Mei P. , Wang X. , Zhang C. , Huang Q. , and Liao Y. , USP7-Mediated ER*β* Stabilization Mitigates ROS Accumulation and Promotes Osimertinib Resistance by Suppressing PRDX3 SUMOylation in Non-Small Cell Lung Carcinoma, Cancer Letters. (2024) 582, 216587, 10.1016/j.canlet.2023.216587, 38097136. 38097136

[34] He Y. , Jiang S. , Zhong Y. , Wang X. , Cui Y. , Liang J. , Sun Y. , Zhu Z. , Huang Z. , and Mao X. , USP7 Promotes Non-Small-Cell Lung Cancer Cell Glycolysis and Survival by Stabilizing and Activating c-Abl, Clinical and Translational Medicine. (2023) 13, no. 12, 10.1002/ctm2.1509, 38082439. PMC10713873 38082439

[35] Goldman M. J. , Craft B. , Hastie M. , Repečka K. , McDade F. , Kamath A. , Banerjee A. , Luo Y. , Rogers D. , Brooks A. N. , Zhu J. , and Haussler D. , Visualizing and Interpreting Cancer Genomics Data via the Xena Platform, Nature Biotechnology. (2020) 38, no. 6, 675–678, 10.1038/s41587-020-0546-8, 32444850. PMC7386072 32444850

[36] Ritchie M. E. , Phipson B. , Wu D. , Hu Y. , Law C. W. , Shi W. , and Smyth G. K. , Limma Powers Differential Expression Analyses for RNA-Sequencing and Microarray Studies, Nucleic Acids Research. (2015) 43, no. 7, e47–e47, 10.1093/nar/gkv007, 2-s2.0-84926507971, 25605792. 25605792 PMC4402510

[37] Xu S. , Hu E. , Cai Y. , Xie Z. , Luo X. , Zhan L. , Tang W. , Wang Q. , Liu B. , Wang R. , Xie W. , Wu T. , Xie L. , and Yu G. , Using clusterProfiler to Characterize Multiomics Data, Nature Protocols. (2024) 19, no. 11, 3292–3320, 10.1038/s41596-024-01020-z, 39019974. 39019974

[38] Feng C. , Song C. , Song S. , Zhang G. , Yin M. , Zhang Y. , Qian F. , Wang Q. , Guo M. , and Li C. , KnockTF 2.0: A Comprehensive Gene Expression Profile Database With Knockdown/Knockout of Transcription (Co-)Factors in Multiple Species, Nucleic Acids Research. (2024) 52, no. D1, D183–D193, 10.1093/nar/gkad1016, 37956336. 37956336 PMC10767813

[39] Wang X. , Li Y. , He M. , Kong X. , Jiang P. , Liu X. , Diao L. , Zhang X. , Li H. , Ling X. , Xia S. , Liu Z. , Liu Y. , Cui C. P. , Wang Y. , Tang L. , Zhang L. , He F. , and Li D. , UbiBrowser 2.0: A Comprehensive Resource for Proteome-Wide Known and Predicted Ubiquitin Ligase/Deubiquitinase–Substrate Interactions in Eukaryotic Species, Nucleic Acids Research. (2022) 50, no. D1, D719–D728, 10.1093/nar/gkab962, 34669962. 34669962 PMC8728189

[40] Xie Y. , Cao Z. , Wong E. W. P. , Guan Y. , Ma W. , Zhang J. Q. , Walczak E. G. , Murphy D. , Ran L. , Sirota I. , Wang S. , Shukla S. , Gao D. , Knott S. R. V. , Chang K. , Leu J. , Wongvipat J. , Antonescu C. R. , Hannon G. , Chi P. , and Chen Y. , COP1/DET1/ETS Axis Regulates ERK Transcriptome and Sensitivity to MAPK Inhibitors, Journal of Clinical Investigation.(2018) 128, no. 4, 1442–1457, 10.1172/jci94840, 2-s2.0-85045061261, 29360641. 29360641 PMC5873878

[41] Keld R. , Guo B. , Downey P. , Gulmann C. , Ang Y. S. , and Sharrocks A. D. , The ERK MAP Kinase-PEA3/ETV4-MMP-1 Axis Is Operative in Oesophageal Adenocarcinoma, Molecular Cancer. (2010) 9, no. 1, 10.1186/1476-4598-9-313, 2-s2.0-78649776481. PMC3009708 21143918

[42] Zhang R. , Peng Y. , Gao Z. , Qian J. , Yang K. , Wang X. , Lu W. , Zhu Y. , Qiu D. , Jin T. , Wang G. , He J. , and Liu N. , Oncogenic Role and Drug Sensitivity of ETV4 in Human Tumors: A Pan-Cancer analysis, Oncology. (2023) 13, 10.3389/fonc.2023.1121258, 37205199. PMC10185867 37205199

[43] Huang L. , Li X. , Huang S. , Jiang Q. , Jiang C. , He W. , Cai Y. , and Guo G. , Comprehensive Analysis of the Functional and Immunological Significance of ETV4 in Pan-Cancer and Its Validation in Digestive Tumors, Frontiers in Immunology. (2025) 16, 10.3389/fimmu.2025.1595850, 40469297. PMC12133824 40469297

[44] Karayel O. , Soung A. , Gurung H. , Schubert A. F. , Klaeger S. , Kschonsak M. , Al-Maraghi A. , Bhat A. A. , Alshabeeb Akil A. S. , Dugger D. L. , and Webster J. D. , Impairment of DET1 Causes Neurological Defects and Lethality in Mice and Humans, Proceedings of the National Academy of Sciences. (2025) 122, no. 7, 10.1073/pnas.2422631122. PMC11848315 39937864

[45] Marine J. C. , Spotlight on the Role of COP1 in Tumorigenesis, Nature Reviews Cancer. (2012) 12, no. 7, 455–464, 10.1038/nrc3271, 2-s2.0-84862763474, 22673153. 22673153

[46] Migliorini D. , Bogaerts S. , Defever D. , Vyas R. , Denecker G. , Radaelli E. , Zwolinska A. , Depaepe V. , Hochepied T. , Skarnes W. C. , and Marine J. C. , Cop1 Constitutively Regulates c-Jun Protein Stability and Functions as a Tumor Suppressor in Mice, Journal of Clinical Investigation. (2011) 121, no. 4, 1329–1343, 10.1172/jci45784, 2-s2.0-79953310717, 21403399. 21403399 PMC3070608

[47] Dornan D. , Wertz I. , Shimizu H. , Arnott D. , Frantz G. D. , Dowd P. , O′ Rourke K. , Koeppen H. , and Dixit V. M. , The Ubiquitin Ligase COP1 Is a Critical Negative Regulator of p53, Nature. (2004) 429, no. 6987, 86–92, 10.1038/nature02514, 2-s2.0-2342447397, 15103385. 15103385

[48] Yoshida A. , Kato J. , Nakamae I. , and Yoneda-Kato N. , COP1 Targets C/EBP*α* for Degradation and Induces Acute Myeloid Leukemia via Trib1, Blood. (2013) 122, no. 10, 1750–1760, 10.1182/blood-2012-12-476101, 2-s2.0-84886461020. 23884858

[49] Li D. Q. , Ohshiro K. , Reddy S. D. N. , Pakala S. B. , Lee M. H. , Zhang Y. , Rayala S. K. , and Kumar R. , E3 Ubiquitin Ligase COP1 Regulates the Stability and Functions of MTA1, Proceedings of the National Academy of Sciences. (2009) 106, no. 41, 17493–17498, 10.1073/pnas.0908027106, 2-s2.0-70350460007, 19805145. PMC2762678 19805145

[50] Suriben R. , Kaihara K. A. , Paolino M. , Reichelt M. , Kummerfeld S. K. , Modrusan Z. , Dugger D. L. , Newton K. , Sagolla M. , Webster J. D. , Liu J. , Hebrok M. , and Dixit V. M. , *β*-Cell Insulin Secretion Requires the Ubiquitin Ligase COP1, Cell. (2015) 163, no. 6, 1457–1467, 10.1016/j.cell.2015.10.076, 2-s2.0-84949255265, 26627735. 26627735

[51] Newton K. , Dugger D. L. , Sengupta-Ghosh A. , Ferrando R. E. , Chu F. , Tao J. , Lam W. , Haller S. , Chan S. , Sa S. , Dunlap D. , Eastham-Anderson J. , Ngu H. , Hung J. , French D. M. , Webster J. D. , Bolon B. , Liu J. , Reja R. , Kummerfeld S. , Chen Y. J. , Modrusan Z. , Lewcock J. W. , and Dixit V. M. , Ubiquitin Ligase COP1 Coordinates Transcriptional Programs That Control Cell Type Specification in the Developing Mouse Brain, Proceedings of the National Academy of Sciences.(2018) 115, no. 44, 11244–11249, 10.1073/pnas.1805033115, 2-s2.0-85055645236, 30322923. PMC6217379 30322923

[52] Gu J. , Xiao X. , Zou C. , Mao Y. , Jin C. , Fu D. , Li R. , and Li H. , Ubiquitin-Specific Protease 7 Maintains c-Myc Stability to Support Pancreatic Cancer Glycolysis and Tumor Growth, Journal of Translational Medicine. (2024) 22, no. 1, 10.1186/s12967-024-05962-6, 39707401. PMC11662425 39707401

[53] Novellasdemunt L. , Foglizzo V. , Cuadrado L. , Antas P. , Kucharska A. , Encheva V. , Snijders A. P. , and Li V. S. W. , USP7 Is a Tumor-Specific WNT Activator for APC-Mutated Colorectal Cancer by Mediating *β*-Catenin Deubiquitination, Cell Reports. (2017) 21, no. 3, 612–627, 10.1016/j.celrep.2017.09.072, 2-s2.0-85032029326, 29045831. 29045831 PMC5656747

[54] Park H. B. , Min Y. , Hwang S. , and Baek K. H. , Suppression of USP7 Negatively Regulates the Stability of ETS Proto-Oncogene 2 Protein, Biomedicine and Pharmacotherapy. (2023) 162, 114700, 10.1016/j.biopha.2023.114700. 37062218

[55] Mevissen T. E. T. and Komander D. , Mechanisms of Deubiquitinase Specificity and Regulation, Annual Review of Biochemistry. (2017) 86, no. 1, 159–192, 10.1146/annurev-biochem-061516-044916, 2-s2.0-85021674924. 28498721

[56] Tracz M. and Bialek W. , Beyond K48 and K63: Non-Canonical Protein Ubiquitination, Cellular and Molecular Biology Letters. (2021) 26, no. 1, 10.1186/s11658-020-00245-6. PMC7786512 33402098

[57] Faesen A. C. , Luna-Vargas M. P. A. , Geurink P. P. , Clerici M. , Merkx R. , van Dijk W. J. , Hameed D. S. , el Oualid F. , Ovaa H. , and Sixma T. K. , The Differential Modulation of USP Activity by Internal Regulatory Domains, Interactors and Eight Ubiquitin Chain Types, Chemistry and Biology. (2011) 18, no. 12, 1550–1561, 10.1016/j.chembiol.2011.10.017, 2-s2.0-84555218153, 22195557. 22195557

[58] Park H. B. , Hwang S. , and Baek K. H. , USP7 Regulates the ERK1/2 Signaling Pathway Through Deubiquitinating Raf-1 in Lung Adenocarcinoma, Cell Death and Disease. (2022) 13, no. 8, 10.1038/s41419-022-05136-6. PMC9365811 35948545

[59] Park J. H. and Chung K. C. , Ubiquitin-Specific Protease 7 Promotes the Growth and Oncogenic Potential of Acute Myeloid Leukemia Cells Through the Deubiquitination and Upregulation of LRRK2, Journal of Biological Chemistry. (2025) 301, no. 10, 110675, 10.1016/j.jbc.2025.110675. 40907896 PMC12510031

[60] Keshri S. , Vicinanza M. , Takla M. , and Rubinsztein D. C. , USP7 Protects TFEB From Proteasome-Mediated Degradation, Cell Reports. (2024) 43, no. 11, 114872, 10.1016/j.celrep.2024.114872, 39412987. 39412987

[61] Bhattacharya S. , Chakraborty D. , Basu M. , and Ghosh M. K. , Emerging Insights Into HAUSP (USP7) in Physiology, Cancer and Other diseases, Therapy. (2018) 3, no. 1, 10.1038/s41392-018-0012-y, 2-s2.0-85058526300, 29967688. PMC6023882 29967688

[62] Xiao J. , Yang S. , Shen P. , Wang Y. , Sun H. , Ji F. , and Zhou D. , Phosphorylation of ETV4 at Ser73 by ERK Kinase Could Block ETV4 Ubiquitination Degradation in Colorectal Cancer, Biochemical and Biophysical Research Communications. (2017) 486, no. 4, 1062–1068, 10.1016/j.bbrc.2017.03.163, 2-s2.0-85017382264, 28373072. 28373072

[63] Pearson A. J. , Fullwood P. , Toro Tapia G. , Prise I. , Smith M. P. , Xu Q. , Jordan A. , Giurisato E. , Whitmarsh A. J. , Francavilla C. , and Tournier C. , Discovery of a Gatekeeper Residue in the C-Terminal Tail of the Extracellular Signal-Regulated Protein Kinase 5 (ERK5), International Journal of Molecular Sciences. (2020) 21, no. 3, 10.3390/ijms21030929, 32023819. PMC7037328 32023819

[64] Lavoie H. , Gagnon J. , and Therrien M. , ERK Signalling: A Master Regulator of Cell Behaviour, Life and Fate, Nature Reviews Molecular Cell Biology. (2020) 21, no. 10, 607–632, 10.1038/s41580-020-0255-7, 32576977. 32576977

[65] Xu Q. , Zhang J. , Telfer B. A. , Zhang H. , Ali N. , Chen F. , Risa B. , Pearson A. J. , Zhang W. , Finegan K. G. , Ucar A. , Giurisato E. , and Tournier C. , The Extracellular-Regulated Protein Kinase 5 (ERK5) Enhances Metastatic Burden in Triple-Negative Breast Cancer Through Focal Adhesion Protein Kinase (FAK)-Mediated Regulation of Cell Adhesion, Oncogene. (2021) 40, no. 23, 3929–3941, 10.1038/s41388-021-01798-2, 33981002. 33981002 PMC8195737

[66] Jiang W. , Cai F. , Xu H. , Lu Y. , Chen J. , Liu J. , Cao N. , Zhang X. , Chen X. , Huang Q. , Zhuang H. , and Hua Z. C. , Extracellular Signal Regulated Kinase 5 Promotes Cell Migration, Invasion and Lung Metastasis in a FAK-Dependent Manner, Protein and Cell. (2020) 11, no. 11, 825–845, 10.1007/s13238-020-00701-1, 32144580. 32144580 PMC7647985

[67] Sánchez-Fdez A. , Re-Louhau M. F. , Rodríguez-Núñez P. , Ludeña D. , Matilla-Almazán S. , Pandiella A. , and Esparís-Ogando A. , Clinical, Genetic and Pharmacological Data Support Targeting the MEK5/ERK5 Module in Lung cancer, Oncology. (2021) 5, no. 1, 10.1038/s41698-021-00218-8, 34404896. PMC8371118 34404896

[68] Zhang C. , Lu J. , Zhang Q. W. , Zhao W. , Guo J. H. , Liu S. L. , Wu Y. L. , Jiang B. , and Gao F. H. , USP7 Promotes Cell Proliferation Through the Stabilization of Ki-67 Protein in Non-Small Cell Lung Cancer Cells, International Journal of Biochemistry & Cell Biology. (2016) 79, 209–221, 10.1016/j.biocel.2016.08.025, 2-s2.0-84984921062, 27590858. 27590858

[69] Shin S. B. , Kim C. H. , Jang H. R. , and Yim H. , Combination of Inhibitors of USP7 and PLK1 Has a Strong Synergism Against Paclitaxel Resistance, International Journal of Molecular Sciences. (2020) 21, no. 22, 10.3390/ijms21228629. PMC7697005 33207738

[70] Malapelle U. , Morra F. , Ilardi G. , Visconti R. , Merolla F. , Cerrato A. , Napolitano V. , Monaco R. , Guggino G. , Monaco G. , Staibano S. , Troncone G. , and Celetti A. , USP7 Inhibitors, Downregulating CCDC6, Sensitize Lung Neuroendocrine Cancer Cells to PARP-Inhibitor Drugs, Lung Cancer. (2017) 107, 41–49, 10.1016/j.lungcan.2016.06.015, 2-s2.0-84977642485, 27372520. 27372520

[71] Peng L. , Zhao Y. , Tan J. , Hou J. , Jin X. , Liu D. X. , Huang B. , and Lu J. , PRMT1 Promotes Warburg Effect by Regulating the PKM2/PKM1 Ratio in Non-Small Cell Lung Cancer, Cell Death & Disease. (2024) 15, no. 7, 10.1038/s41419-024-06898-x, 39009589. PMC11251085 39009589

