# Supplementary material for: Deubiquitination of ETV4 by USP7 Promotes NSCLC Tumorigenesis via MAPK7 Activation
Source: Hum Mutat. 2026 May 6;2026:9432303. doi: 10.1155/humu/9432303 (PMC13147211; doi:10.1155/humu/9432303)
Supplement: Supplementary file 1 — Supporting Information 1 Supporting materials and methods. [file HUMU-2026-9432303-s003.docx]

**Supplementary Material and Methods**

siRNA transfection, plasmid construction, and transfection, generation of stable cells

The siRNAs specifically targeting ETV4, USP7, MAPK7, and control siRNA were synthesized by GenePharma (Shanghai, China). The siRNA sequences can be found in Table S2, Supporting Information. pcDNA3.1-MAPK7 plasmids (EX-T8074-M11, neomycin) were synthesized by Genepharma (Shanghai, China). The full-length of Flag-ETV4 plasmids (EX-T8074-M11, neomycin) were obtained from GeneCopoeia (Guangzhou, China). **S**h-ETV4 lentiviral vectors (LV3, Puro) or negative control (NC) shRNA were obtained from GenePharma (Shanghai, China). Transfections were performed using Lipofectamine 2000 reagent (Invitrogen, Grand Island, NY) following the manufacturer’s protocol. A549 cells transfected with ETV4 or NC shRNA vectors were selected with puromycin to obtain stably ETV4-depleting (A549 sh-ETV4) clones. Besides, A549 sh-ETV4 cells were transfected with MAPK7 or empty vectors and selected with both neomycin and puromycin to obtain sh-ETV4+vector or sh-ETV4+OE-MAPK7 stable cells.

RNA extraction, cDNA synthesis, and RT-qPCR

Total RNA from cultured cells was extracted using TRIzol reagent (Invitrogen). The first-strand cDNA was first synthesized from 1 μg RNA using FastKing Reverse Transcription Kit (Tiangen, China), followed by PCR amplification using 5% cDNA for each reaction on the Agilent Mx3005P QPCR System. ACTB gene was used as an internal control. The expression of each target gene was calculated using the 2^−ΔΔCt^ method and presented as relative mRNA expression. Experiments were repeated at least three times. The respective primers can be found in Table S2, Supporting Information.

Western blot analysis

Cells were lysed on ice for 30 min in RIPA lysis buffer supplemented with a protease inhibitor cocktail and a phosphatase inhibitor. The protein concentration was measured with BCA Protein Assay Kit (Solarbio, China). Equal amounts of total protein were separated by SDS‐PAGE on 10% gels and transferred onto polyvinylidene fluoride (PVDF) membranes (Millipore). After blocking with 5% skim milk for 1h at room temperature, the membranes were incubated with antibodies specific for ETV4 (10684-1-AP, Proteintech), USP7 (4833, CST), ERK5 (3372, CST), Flag (14793, CST), Ub (1106RM, PTM Bio) at 4°C overnight, and β-actin (66009-1-Ig, Proteintech) was used as a loading control for Western blot. The blots were then re-probed with a secondary antibody, visualized by the chemiluminescence and scanned using ImageQuant LAS 4010 Imaging System (GE Healthcare Life Sciences, Piscataway, NJ).

**Chromatin immunoprecipitation (ChIP) assay**

The interaction of the ETV4 with the MAPK7 gene promoter was analyzed using the ChIP Assay Kit (Merck Millipore, USA) following the manufacturer’s protocol. Briefly, cells were subjected to 1% formaldehyde incubation for 10 min to cross-link DNA and its interacting proteins. The cross-linked DNA-protein complexes were then sheared into ~500 bp DNA fragments using shearing by sonication, immunoprecipitated by a Flag antibody (14793, CST) for exogenous ETV4 or ETV4 antibody (sc-113x, Santa Cruz) for endogenous ETV4 detection, or IgG (2729, CST) as a control. After the cross-links were reversed, the precipitated chromatin DNA was eluted, followed by (q)PCR analysis. Primers used for MAPK7 gene promoter ChIP-(q)PCR assays were listed in

Table S2, Supporting Information.

Luciferase reporter assay

To create MAPK7 gene promoter luciferase reporter constructs, 1247 bp fragments of promoter DNA were synthesized and cloned into the pEZX-FR01 luciferase report vector (GeneCopoeia, Guangzhou, China). The plasmid of pEZX-FR01-MAPK7 (Luc-pEZX-MAPK7) was constructed in our lab. The primers used for Luciferase reporter construction can be found in Table S2, Supporting Information. The amplified fragment was cloned into the pEZX-FR01 vector at MluI and BamHI sites. The plasmid sequence was confirmed by DNA sequencing. Luc-pEZX-MAPK7 plasmids were co-transfected with ETV4 plasmid or the empty vector into H358 or HEK293T cells. The luciferase activity was measured using the Luc-Pair™ Duo-Luciferase HS Assay Kit (GeneCopoeia) and normalized to Renilla luciferase activity. Experiments were repeated at least thrice.

**Proximity Ligation Assay (PLA)**

PLA was performed using the Duolink® Proximity Ligation Assay (DUO92101, Sigma) following the manual provided by manufacturer. 1×10⁶ cells were plated in 6-well plates overnight. Then cells were fixed with 4% paraformaldehyde solution, permeabilized using 0.5% Triton-X100, and blocked with the provided blocking solution and subsequently probed with mouse-anti-ETV4 antibody (sc-113, Santa Cruz) and rabbit-anti-USP7 antibody (4833, CST) at 4°C overnight. Detection was performed using species-specific PLA probes anti-mouse PLUS and anti-rabbit MINUS (diluted 1:5 in the provided buffer), with incubation at 37°C for 1h. Following three 5-min washes in Buffer A, the ligation reaction was proceeded for 30 min at 37°C. Signal amplification was achieved through incubation for 100 min at 37°C after two brief Buffer A washes. Final washing steps included three 10-min washes in Buffer B and a 1-min wash in diluted Buffer B (0.01×). Cells were mounted with DAPI-containing medium. Fluorescent signals were visualized using Leica Laser Confocal Microscope (Leica, Germany) with 60×objective oil immersion.

Cell proliferation assays

4×10^3^ cells were seeded onto 96-well plates in standard culture condition overnight, followed by siRNA or plasmid transfection. The cell viability was assessed in six replicates at 24, 48, 72, and 96 h after transfection by MTT assay. The experiments were performed at least three times.

Clonogenic assay

Cells were seeded at a 6-well plate 48 hours after siRNA or plasmid transfection. 14 days after treatment, colonies were fixed with 3% paraformaldehyde for 10 minutes and stained with 0.1% crystal violet solution within 30 minutes. The number of colonies was counted and normalized to the control cells. All experiments were conducted in triplicate replicates.

**EdU assay for Confocal microscope test**

EdU (5-ethynyl-2’-deoxyuridine), a nucleoside analog of thymidine, is readily incorporated into cellular DNA during DNA replication. Cell proliferation was evaluated using a Cell-Light EdU Apollo 488 In Vitro Imaging Kit (Ribobio, Guangzhou, China) as described by the manufacturer. Briefly, cells were previously seeded onto coverslips and incubated overnight. 48h post transfection, cells were incubated with 50 μM EdU for 2 h at 37°C, fixed with 4% formaldehyde, then stained with Apollo reaction cocktail and Hoechst 33342, protected from light. Images were acquired with a Leica Laser Confocal Microscope (Leica, Germany).

**Immunohistochemistry assay**

Paraffin-embedded lung tissues were incubated with the following antibodies: anti-ETV4 antibody (10684-1-AP, Proteintech), anti-USP7 antibody (4833, CST), and anti-ERK5 antibody (3372, CST). Immunohistochemical scoring of nuclear staining of ETV4 and USP7 was performed as follows: positive nuclear expression in over 50% of the tumor cells was scored as positive. Cytoplasmic staining of ERK5 protein was quantified based on a multiplicative index of staining extent (0‐3) and the average staining intensity (0‐3). Quantification of the three proteins’ expression was performed blindly by a pathologist.
