# Supplementary material for: Deubiquitination of ETV4 by USP7 Promotes NSCLC Tumorigenesis via MAPK7 Activation
Source: Hum Mutat. 2026 May 6;2026:9432303. doi: 10.1155/humu/9432303 (PMC13147211; doi:10.1155/humu/9432303)
Supplement: Supplementary file 2 — Supporting Information 2 Additional results (Figures S1–S5). [file HUMU-2026-9432303-s002.docx]

**Supplementary Figure**


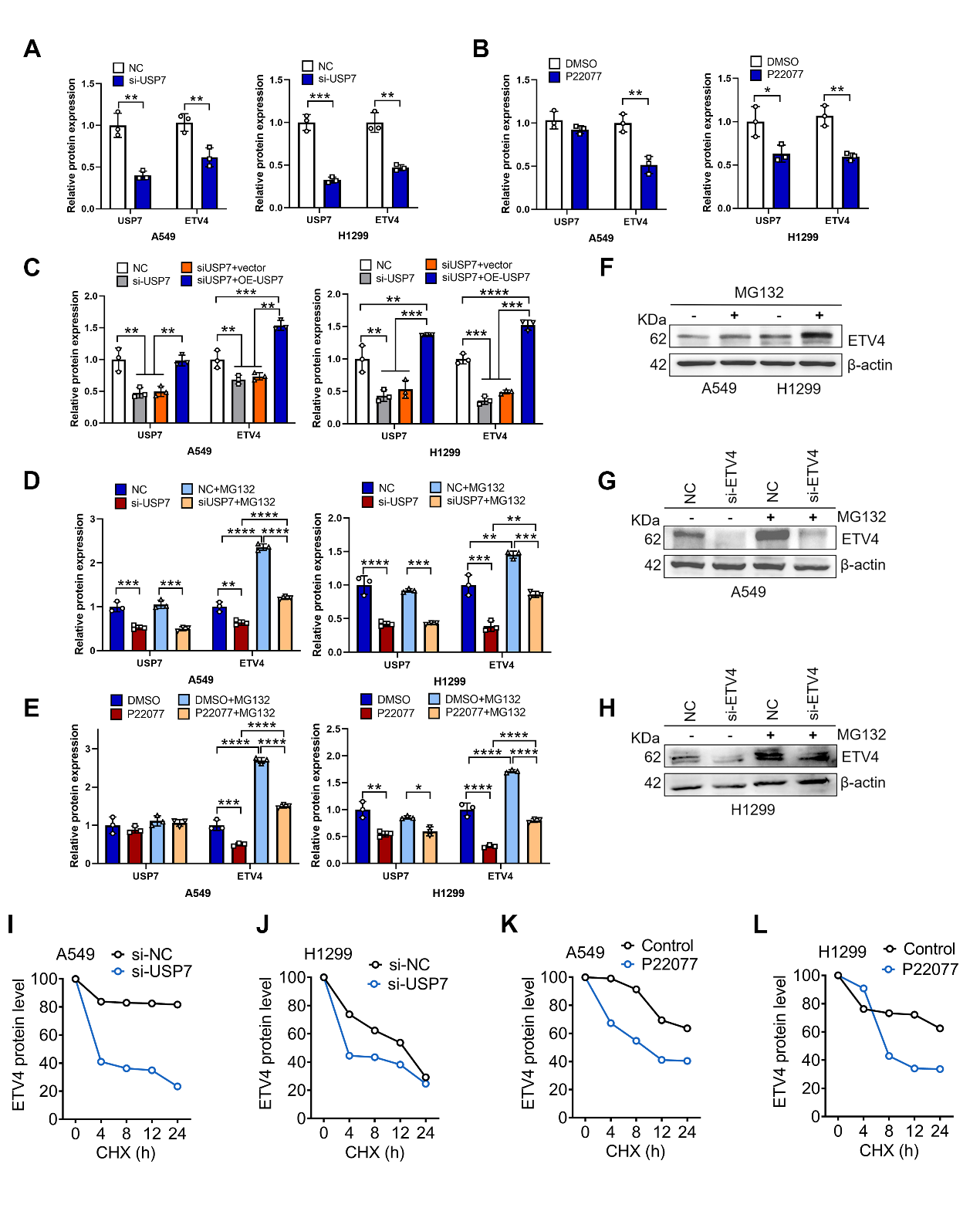


**Figure S1. USP7 maintains ETV4 protein stability in NSCLC Cells.**

**(A-E)** Quantitative analysis of Western blot results from Figure 3A-E. Data are presented as mean±SD from three independent experiments (n = 3). Statistical significance was determined by two-tailed unpaired *t*-test or One-way ANOVA followed by Tukey’s multiple comparisons test. **P* < 0.05, ***P* < 0.01, ****P* < 0.001, *****P* < 0.0001. **(F)** ETV4 expression in A549 and H1299 cells treated with 20 μM MG132 for 24 h. **(G,H)** ETV4 protein expression in A549 and H1299 cells transfected with NC or ETV4 siRNA, with or without MG132 treatment. **(I-L)** Quantification of Western blot data in (Figure 3F-I) relative to control (0 h).


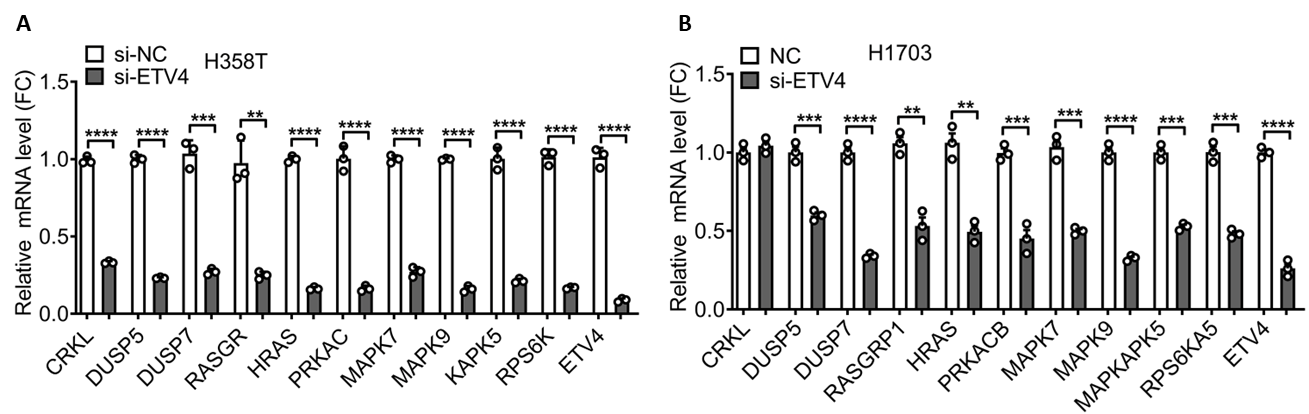


**Figure S2. RT-qPCR validation of 10 MAPK pathway genes in ETV4-deficient NSCLC cells.**

**(A-B)** RT-qPCR validation of 10 MAPK pathway genes in ETV4-deficient H358T and H1703 cells. Transcript levels were normalized to ACTB gene expression (mean ± SD, n = 3; two-tailed unpaired t-test). **P < 0.01; ***P < 0.001; ****P < 0.0001.


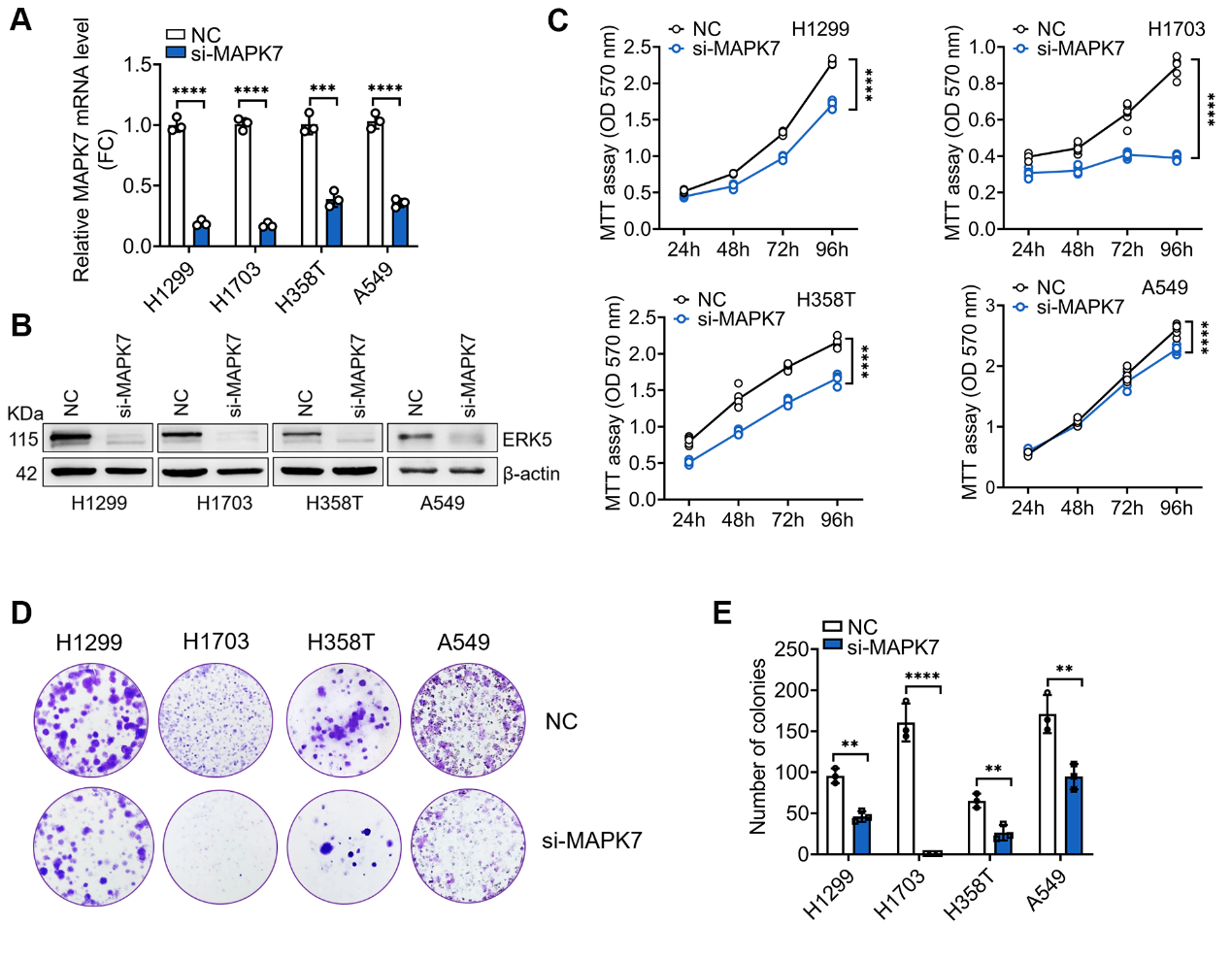


**Figure S3. MAPK7 contributes to cell proliferation in NSCLC cells.**

**(A)** MAPK7 mRNA expression in H1299, H1703, H358T and A549 cells transfected with NC or si-MAPK7 (50 nM, 48 h) using RT-qPCR assay (mean ± SD, n = 3; two-tailed unpaired *t*-test). ****P* < 0.001; *****P* < 0.0001. **(B)** ERK5 protein level in NC and MAPK7 knockdown cells. **(C)** Cell viability of H1299, H1703, H358T, and A549 cells in the indicated periods (0 - 96 h) after transfection with NC or si-MAPK7. (mean ± SD, n = 6; Two-way ANOVA with Bonferroni’s multiple comparisons test). *****P* < 0.0001. **(D, E)** Colony formation assays in H1299, H1703, H358T, and A549 cells transfected with NC or si-MAPK7 (mean ± SD, n = 3; two-tailed unpaired *t*-test). ***P* < 0.01; *****P* < 0.0001.


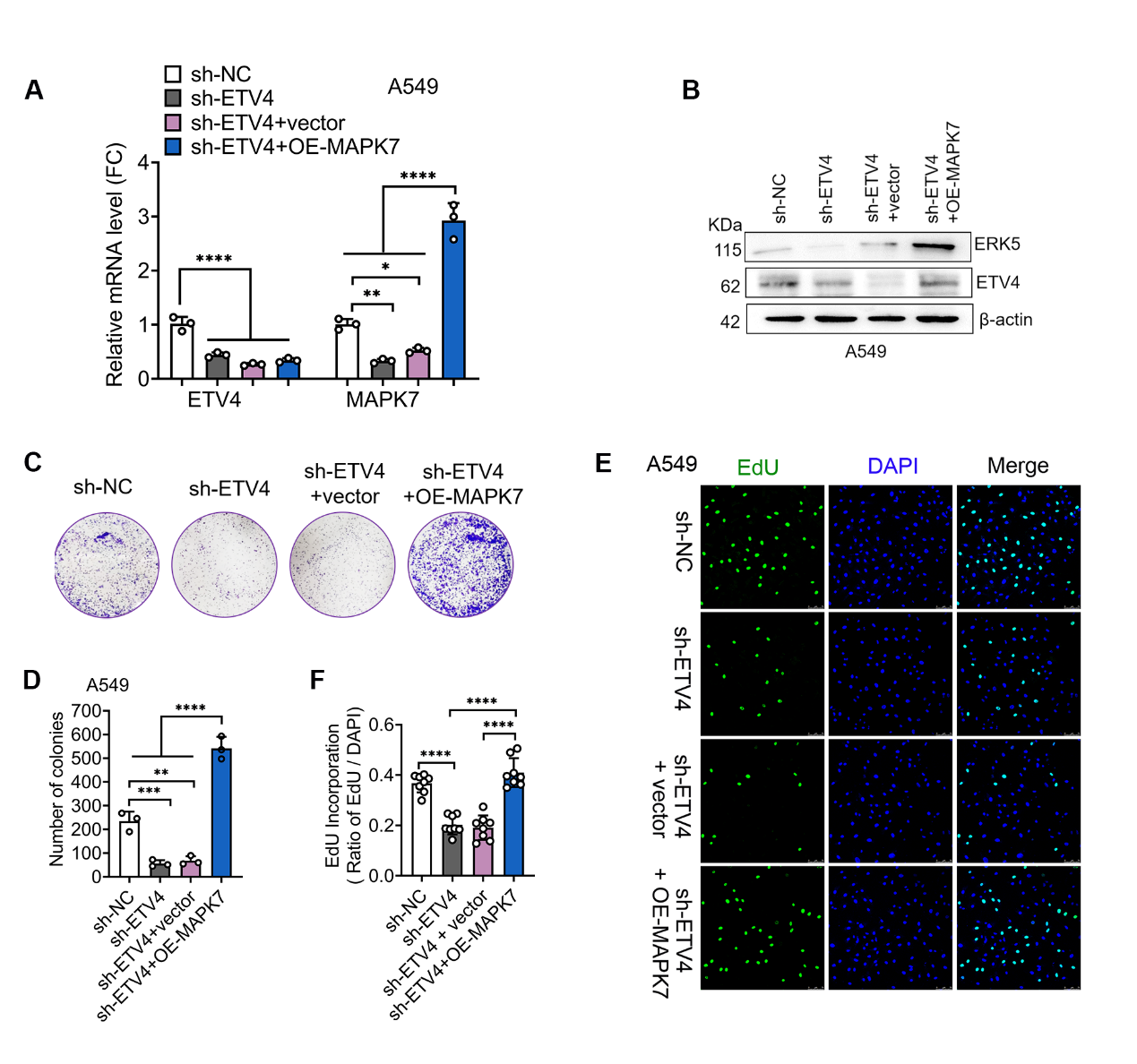


**Figure S4.** **MAPK7 overexpression reverses the proliferation defect caused by ETV4 knockdown.**

**(A)** A549‑shETV4 cells stably overexpressing MAPK7 were selected using neomycin and puromycin. mRNA levels of ETV4 and MAPK7 were measured by RT-qPCR (mean ± SD, n = 3; One-way ANOVA followed by Tukey’s multiple comparisons test). **P* < 0.05; ***P* < 0.01; *****P* < 0.0001. **(B)** ETV4 and ERK5 protein levels in A549 sh-NC, sh-ETV4, sh-ETV4+vector, and sh-ETV4+OE-MAPK7 cells, respectively. **(C, D)** Colony formation assays showing the effects of MAPK7 overexpression in A549-shETV4 cells (mean ± SD; n = 3; One-way ANOVA followed by Tukey’s multiple comparisons test). ***P* < 0.01; ****P* < 0.001; *****P* < 0.0001. (**E, F)** EdU-incorporation analysis of MAPK7 overexpression in A549-shETV4 cells (mean ± SD; n = 8; One-way ANOVA followed by Tukey’s multiple comparisons test). *****P* < 0.0001.


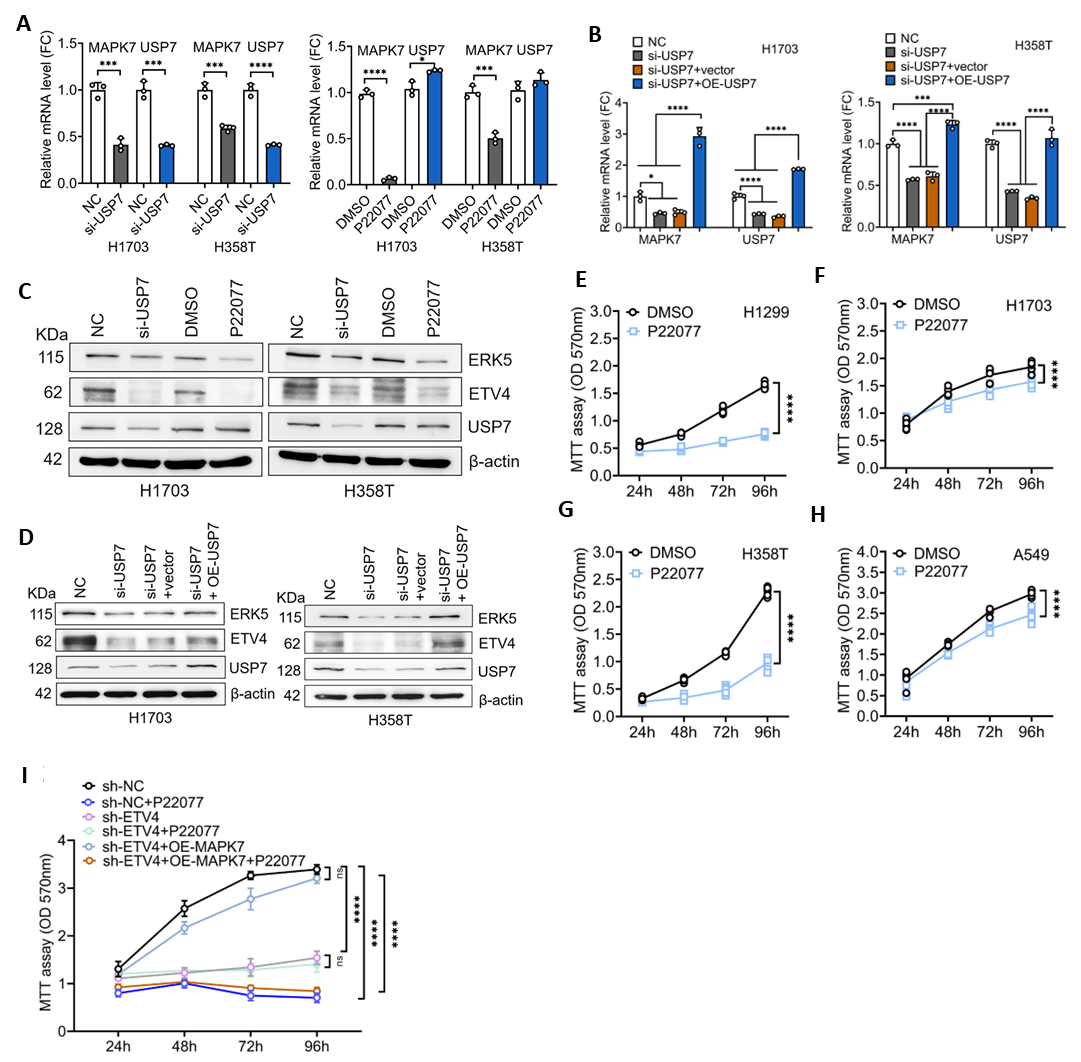


**Figure S5. P22077 inhibits ETV4-MAPK7 induced NSCLC cell proliferation in vitro and tumor growth and in vivo.**

**(A)** RT-qPCR analysis of MAPK7 and USP7 expression in NSCLC cells after USP7 knockdown (si-USP7) or pharmacological inhibition (P22077, 50 μM, 6 h) (mean ± SD, n = 3; two-tailed unpaired t-test). ***P < 0.001; ****P < 0.0001. **(B)** RT-qPCR analysis of MAPK7 and USP7 transcript levels under USP7 knockdown and rescue conditions, with normalization to ACTB expression (mean ± SD; n = 3; One-way ANOVA followed by Tukey’s multiple comparisons test). ****P < 0.0001. *P < 0.05; **P < 0.01; ****P < 0.0001. **(C)** Western blot analysis of ETV4 and ERK5 protein expression following genetic or pharmacological USP7 suppression in multiple NSCLC cell lines. **(D)**Western blot detection of ETV4 and ERK5 protein levels upon USP7 reconstitution in USP7-deficient cells. **(E-H)** Cell proliferation of H1299, H1703, H358T, and A549 cells in the indicated periods (0 - 96 h) after treatment with P22077 at a concentration of 10 μM. (mean ± SD, n = 6; Two-way ANOVA with Bonferroni’s multiple comparisons test). ****P < 0.0001. **(I)** Cell proliferation of A549 sh-NC, sh-ETV4, and sh-ETV4+OE-MAPK7 cells after treatment with P22077 (10μM).
