# Supplementary material for: Deubiquitination of ETV4 by USP7 Promotes NSCLC Tumorigenesis via MAPK7 Activation
Source: Hum Mutat. 2026 May 6;2026:9432303. doi: 10.1155/humu/9432303 (PMC13147211; doi:10.1155/humu/9432303)
Supplement: Supplementary file 3 — Supporting Information 3 Table S1 showing relationships between ETV4, USP7, and ERK5 expression and clinicopathologic parameters. [file HUMU-2026-9432303-s001.docx]

**Table S1.** Relationships between ETV4, USP7, ERK5 expression and clinicopathologic parameters in 77 NSCLCs

| **Characteristics** | **cases** | **ETV4** | | ***P***  **value** | **USP7** | | ***P***  **value** | **ERK5** | | ***P***  **value** |
| --- | --- | --- | --- | --- | --- | --- | --- | --- | --- | --- |
|  |  | **+** | **-** |  | **+** | **-** |  | **+** | **-** |  |
| **All cases** | 77 | 43 | 34 |  | 41 | 36 |  | 42 | 35 |  |
| **Age (y)** |  | | | 0.862 |  | | 0.365 |  | | 0.280 |
| <=60 | 28 | 16 | 12 |  | 13 | 15 |  | 13 | 15 |  |
| >60 | 49 | 27 | 22 |  | 28 | 21 |  | 29 | 20 |  |
| **Sex** |  | | | 0.562 |  | | 0.902 |  | | 0.220 |
| Male | 54 | 29 | 25 |  | 29 | 25 |  | 27 | 27 |  |
| Female | 23 | 14 | 9 |  | 12 | 11 |  | 15 | 8 |  |
| **Histological type** |  | | | 0.472 |  | | 0.683 |  | | 0.379 |
| LUAD | 51 | 27 | 24 |  | 28 | 23 |  | 26 | 25 |  |
| LUSC | 26 | 16 | 10 |  | 13 | 13 |  | 16 | 10 |  |
| **Tumor size** |  | | | 0.026 |  | | 0.010 |  | | 0.006 |
| <=3cm | 27 | 10 | 17 |  | 9 | 18 |  | 10 | 18 |  |
| >3cm | 50 | 33 | 17 |  | 32 | 18 |  | 33 | 17 |  |
| **Lymphnode metastasis** |  | | | 0.037 |  | | 0.061 |  | | 0.084 |
| Negative | 54 | 26 | 28 |  | 25 | 29 |  | 26 | 28 |  |
| Positive | 23 | 17 | 16 |  | 16 | 7 |  | 16 | 7 |  |
| **Metastasis** |  | | | 0.021 |  | | 0.050 |  | | 0.016 |
| No | 61 | 30 | 31 |  | 29 | 32 |  | 29 | 32 |  |
| Yes | 16 | 13 | 3 |  | 12 | 4 |  | 13 | 3 |  |
| **Stage** |  | | | 0.010 |  | | 0.035 |  | | 0.016 |
| I | 33 | 11 | 22 |  | 13 | 20 |  | 13 | 20 |  |
| II-IV | 44 | 32 | 12 |  | 28 | 16 |  | 29 | 15 |  |

ETV4: ETS variant transcription factor 4; ERK5: extracellular signal-regulated kinase; MAPK7: mitogen-activated protein kinase 7; USP7: ubiquitin-specific protease 7; NSCLC: non-small cell lung cancer; LUAD: lung adenocarcinomas; LUSC: lung squamous cell carcinoma.
