# Supplementary material for: Deubiquitination of ETV4 by USP7 Promotes NSCLC Tumorigenesis via MAPK7 Activation
Source: Hum Mutat. 2026 May 6;2026:9432303. doi: 10.1155/humu/9432303 (PMC13147211; doi:10.1155/humu/9432303)
Supplement: Supplementary file 4 — Supporting Information 4 Table S2 listing siRNA and primer sequences. [file HUMU-2026-9432303-s004.docx]

Table S2. Primers used for siRNA, RT-qPCR, ChIP-(q)PCR, and luciferase reporter construction in this study.

| siRNA sequences | | |
| --- | --- | --- |
| Gene | Forward (5’-3’) | Reverse (5’-3’) |
| ETV4 | CCAGACAAAUCGCCAUCAATT | UUGAUGGCGAUUUGUCUGGTT |
| USP7 | AUAGUGAUAAACCUGUAGGTT | CCUACAGGUUUAUCACUAUGC |
| MAPK7 | CAAGUACCAUGAUCCUGAUTT | AUCAGGAUCAUGGUACUUGGC |
| Negative control | UUCUCCGAACGUGUCACGUTT | ACGUGACACGUUCGGAGAATT |
| Primers for RT-qPCR | | |
| Gene | Forward (5’-3’) | Reverse (5’-3’) |
| ETV4 | GAAGGAGACATCAAGCAGGAA | AGCAAGGCCACCAGAAAT |
| MAPK7 | CAAATCTGTCTACGTGGTCCTG | CAGCAGTTGGTACAGAAGTAG |
| CRKL | CTTTGCCCACACAGAATG | CACGATGTCACCAACCTCTAA |
| DUSP5 | GCGGGTCTACTTCCTCAAAG | GGCTCTCTCACTCTCAATCTTC |
| DUSP7 | GAGTTCACCTACAAGCAGATCC | CACCAGGACACCACACTTC |
| **RASGRP1** | CAACTTCCAAGAGACCACCTAC | ATGTCATCCCGCAGTCTTTAC |
| **HRAS** | GGAATATAAGCTGGTGGTGGG | GCTTCCGGTAGGAATCCTCTAT |
| PRKACB | TCTCAGCAAGGGCTACAATAAG | ATTGGTTGGTCTGCAAAGAATG |
| MAPK9 | GGGTATGGGCTACAAAGAGAAC | ATGGTCAGTGCCTTGGAATATC |
| MAPKAPK5 | CAGAAGGAGAAATCTGGCATCA | ATCCGCACAGCATCACATAG |
| RPS6KA5 | CATGCTGAGAAGGTGGGAATAG | GGCATACAGCTTTCCAGTATCA |
| ACTB | AGCGAGCATCCCCCAAAGTT | GGGCACGAAGGCTCATCATT |
| Primers for anti-ETV4 ChIP-(q)PCR (gene promoter) | | |
| **MAPK7 ChIP-PCR** | TCCGCAGAGGAGCAGAGGTTGGG | CCTCCACTGACTTCCGGTGGCTGAG |
| **MAPK7 ChIP-qPCR** | GCAGAGGAGCAGAGGTTG | GGTTTCCTGGCTTCTAGGTATC |
| Primers for luciferase reporter construction | | |
| **Luc-MAPK7 promoter** | CGACGCGTTAGGTCTCCACAACCCCTTATCAT (Mlu) | CGCGGATCCTTGGCTACTGGCTCCCGAA (BamH I） |

ACTB**:** actin beta; CRKL: CRK like proto-oncogene; ChIP: chromatin immunoprecipitation; DUSP5: dual specificity Phosphatase 5; DUSP7: dual specificity Phosphatase 7; ETV4: ETS variant transcription factor 4; HRAS: HRas proto-oncogene; MAPK7: mitogen-activated protein kinase 7; MAPK9: mitogen-activated protein kinase 9; MAPKAPK5: MAPK activated protein kinase 5; RASGRP1: RAS guanyl releasing protein 1; PRKACB: protein kinase cAMP-activated catalytic subunit beta; RPS6KA5: ribosomal protein S6 kinase A5; RT-(q)PCR: real-time (quantitative) PCR; USP7: ubiquitin-specific protease 7.
